# To what extent does Together with Gloria! expand the reach of *SASA! Together* community programming? A mixed methods evaluation of an edutainment intervention in Kasese, Uganda

**DOI:** 10.1186/s12889-025-24178-x

**Published:** 2025-09-29

**Authors:** Tanya Abramsky, Shaffa Hameed, Junior Brian Musenze, Alice Witt, Herbert Mugooda, Dan Opito, Esther Awino, Gabriella Pinto, Sophie Namy, Janet Nakuti, Ana Maria Buller

**Affiliations:** 1 Department of Global Health and Development, London School of Hygiene and Tropical Medicine, 15-17 Tavistock Place, London, WC1H 9SH UK; 2 Raising Voices, 16 Tufnell Drive, Kamwokya, P.O Box 6770, Kampala, Uganda; 3 Research World International Ltd, RWI House, Off Lusirika Road, Kira, Uganda; 4 MRC/UVRI and LSHTM Uganda Research Unit, Plot No: 51 -59 Nakiwogo Road, P.O.Box 49, Entebbe, Uganda

**Keywords:** Intimate partner violence, Violence against women, Violence prevention, Edutainment, Mass-media, Community interventions, Social norms, Gender norms, Uganda, Africa

## Abstract

**Background:**

Community-level interventions to prevent violence against women (VAW), though potentially efficacious, are challenging to implement at scale, requiring intensive long-term engagement. There is thus interest in the potential for mass-media strategies, including ‘edutainment’, to complement and extend the reach of in-person community programming. While edutainment interventions can demonstrably reach large audiences at relatively low cost, little is known about who they reach/exclude, nor about barriers/facilitators to engagement. This paper describes the extent to which ‘Together with Gloria!’ (TWG), a 33-episode radio-drama, expanded the reach of *SASA! Together,* a community-level VAW prevention intervention in Western Uganda.

**Methods:**

As part of a broader mixed-methods evaluation, we conducted two cross-sectional community surveys: baseline (6-weeks into broadcasting); and endline (6-weeks after final broadcast). Qualitative data collection included focus group discussions and in-depth interviews with community members, local leaders and *SASA! Together* Community Activists. We present descriptive data on TWG’s reach, and the extent to which people listened/discussed TWG with others. We use logistic regression to explore variation in reach by socio-demographic characteristics. Barriers/facilitators to engagement are explored using survey and qualitative data.

**Results:**

At endline (*n* = 1018), 52% of survey respondents reported having listened to TWG. Across all demographic sub-groups, exposure to TWG considerably exceeded exposure to in-person *SASA! Together* programming, with TWG listening more uniformly spread across villages. Among men, listening was positively associated with increasing age, having a partner and children, and radio ownership. Among women, listening was positively associated with secondary education, being childless, and owning a radio. Most listeners (74% men; 64% women) reported usually listening with others (most commonly a partner), and 41% had discussed TWG with other community members. Communal listening and multiple broadcast stations/timeslots were noted to facilitate listening. Barriers included lack of radio access (particularly among women), and lack of time.

**Conclusions:**

TWG expanded overall reach of in-person *SASA! Together* programming, and reduced geographical disparities in access. Use of multiple radio-stations/broadcasting timeslots allowed many to listen despite competing time-commitments. Future revisions to broaden access and appeal could include communal broadcasts in public places, and the incorporation of characters and storylines to resonate with younger childless men.

**Supplementary Information:**

The online version contains supplementary material available at 10.1186/s12889-025-24178-x.

## Background

Violence against women (VAW) is a major global public health, human rights and development concern. Globally, an estimated 27% of ever-partnered women aged 15 to 49 years have experienced physical and/or sexual intimate partner violence (IPV) during their lifetime [1]. Violence begins early, with almost a quarter of 15–19 year-old girls already subjected to IPV at least once in their lives [2]. The consequences of VAW are severe and far-reaching, impacting on women’s physical and mental health [3–5], the health of their children and families, and the likelihood that their children later go on to experience and/or perpetrate violence themselves [6, 7].

The past decade has seen a rapid growth in research on VAW and violence prevention programming [8]. This has included growing recognition of the need to work with entire communities to shift the knowledge, attitudes, behaviours and social norms around gender, power and VAW that underpin high community levels of VAW [9–11]. One community-level prevention approach that has received considerable attention is community mobilisation. Using trained community activists, such interventions seek to engage entire communities—ordinary men and women, community and cultural leaders, and service providers – to challenge harmful norms and behaviours that perpetuate VAW and hinder community-level responses [8].

These multi-faceted community-level interventions, while demonstrated in several rigorous evaluations to be an effective means of VAW prevention [12–16], are challenging to implement, typically requiring intensive engagement over a long period of time. Recent reviews of the evidence suggest that the success of such approaches depends on very strong design and implementation, intensive facilitator training and oftentimes extensive volunteer time [8, 17], making scale-up of such programs particularly challenging. There is concern too over how to maximise inclusion of ‘harder to reach’ communities, such as those that are sparsely populated or geographically remote, as well as those affected by conflict, unrest or humanitarian crises. Within communities, individual-level factors can also impede programme reach to, for example, those affected by disability or those less able to leave the house because of social, economic or cultural constraints. The COVID-19 pandemic, and the ensuing lockdowns and restrictions on public gathering, have highlighted such challenges on a global scale. During this time, many women in violent relationships found themselves confined to their homes alongside violent partners, at the same time as VAW prevention and response programming suffered unprecedented levels of disruption [18]. There has thus been renewed impetus for the VAW prevention field to find new ways to engage communities effectively and safely. This includes exploring the role that mass media could play in complementing and expanding the reach of violence prevention programming.

Campaigns that use mass media communication methods such as television or radio are attractive as they can reach large numbers of people at relatively low cost. They aim to raise awareness about issues and services, encourage service uptake, provoke discussion, discourage harmful norms and promote positive norms [19]. Evidence from a range of fields has shown the impact that such interventions may have on a diverse array of health knowledge, attitude and behaviour outcomes [20–25]. Increasingly, the potential of edutainment – the theory-based embedding of education on social issues within entertainment programmes—to promote individual, community, institutional and societal changes has been recognised [19, 26]. In addition to their mass reach, such programmes might instigate mechanisms of change less tapped into by other forms of intervention. Existing examples have drawn on a multitude of theoretical approaches, with a strong influence from Bandura’s social cognitive theory (SCT) and social learning theory (SLT) [27, 28] – emphasising how people learn behaviour from their environment through observation, imitation and modelling. These include the widely adopted Sabido method [29, 30] as well as more recently articulated theory such as that of ‘transportation’ [31, 32] which posits absorption into the story world as a key element in narrative persuasion and the generation of social, normative and behavioural change.

The evidence-base around edutainment as a means to prevent VAW is limited, though has received increased attention in recent years. The role of media in multicomponent interventions has been associated with positive impacts on IPV knowledge and awareness, increased willingness to disclose IPV, decreases in gender-inequitable attitudes, greater joint decision-making and improved communications around domestic violence and sexual decision-making [33–38]. Examples of more comprehensive IPV edutainment campaigns include Soul City in South Africa [33] and Puntos de Encuentro’s Sexto Sentido in Nicaragua [34], which integrated social messages into high-quality popular radio and television drama series/talk shows. Despite these results, doubts remain about the potential for media interventions alone to lead to sustained change in community-levels of IPV because of their didactic one-way nature [10]. Furthermore, few such interventions have been subject to rigorous evaluations, and evaluations to date have produced heterogeneous findings [38]. A recent review of interventions to prevent IPV concluded that such interventions ‘may have a role in combination with other components of interventions designed to impact at a community level’ [8], as was the case with Soul City and Sexto Sentido which were embedded within broader community mobilisation activities.

While evidence (and common sense) indicates that such interventions can indeed reach large numbers of people at relatively low cost [34, 39], little is known about who edutainment interventions (across a range of topics) reach and conversely ‘exclude’. Evaluations to date have been conducted disproportionately among urban comparatively more educated populations [40]. With few exceptions [25], scant data exist on how reach differs according to socio-demographic characteristics, which factors facilitate or act as barriers to listening among different sub-groups, and the means through which people might overcome these barriers. We also lack evidence on the structure of people’s engagement with edutainment interventions, important for our understanding of the mechanisms through which such interventions may promote change. For example, evidence is lacking on the extent to which people engage with such interventions communally (potentially important in the establishment of ‘new’ norms) or discuss programme content/messaging/learning with others in their community (‘social diffusion’) [38]. Furthermore, few studies [33] have explored interactions and overlap in reach between edutainment and face to face programming, an important evidence gap since edutainment has been widely touted as an intervention best used in combination with other community-level intervention components.

In response to current challenges and evidence gaps, we created and evaluated the *SASA! Together* radio drama (Together with Gloria!), implemented alongside the *SASA! Together* community mobilisation intervention in Kasese District in Western Uganda. The *SASA!* intervention [41] (an earlier iteration of *SASA! Together*) was the first community-mobilisation intervention shown, in a cluster randomised trial in Kampala, Uganda (2014), to have community-level impacts on IPV and related attitudes [12, 13]. ‘Together with Gloria!'(TWG), a 33-episode weekly radio show, was developed to: 1) circumvent community- and individual-level barriers to in-person programming/participation, thereby expanding the reach of *SASA! Together* community programming; and 2) boost the effects of in-person *SASA! Together* programming both by increasing participation and reinforcing/bringing to life programme messaging and ideas.

This paper explores the extent to which TWG expanded the reach of *SASA! Together*, and the nature in which it did so. We use quantitative data from cross-sectional surveys of community members to estimate the extent and patterns of TWG listening, identify individual- and community-level factors associated with listening, and explore overlap between TWG listening and exposure to in-person *SASA! Together* programming. We also report on the extent to which listeners have discussed TWG with others in the community. Alongside the quantitative survey data, we use qualitative longitudinal data from community members, activists and leaders, to explore facilitators and barriers to TWG listening. Potential ‘booster effects’ of TWG will be explored in a separate paper.

## Methods

### Setting

The study assessed the implementation of the *SASA! Together* radio drama alongside *SASA! Together* community programming in Kisinga Sub County of Kasese District in Western Uganda. Kisinga Sub County comprises 6 parishes and 37 villages, and is largely rural. While some villages bear features of small towns and have access to amenities such as electricity, piped water supplies, improved housing and trading centres (thus classed as urban), some of the rural villages are extremely geographically remote and not easily accessible by road. The District of Kasese as a whole is predominantly agricultural, with most households engaged in subsistence farming.

The population of Kasese District is young, with over half of the inhabitants under 18 years of age [42]. Though there are three tribes indigenous to Kasese District, the vast majority of the population in Kisinga Sub County belong to the Bakonzo tribe and speak the Lukhonzo language. An estimated 58% of 15–49 year old ever-married women in the region have experienced physical, sexual or emotional IPV during their lifetime, with acts of economic abuse and controlling behaviours by a partner also commonplace [43]. Patriarchal norms are prevalent, with 40% of women and 42% of men expressing the belief that it is acceptable for a husband to use violence against his wife [43]. The pervasive nature of non-partner violence against women, oftentimes perpetrated by other family members, was also highlighted by a recent report into violence in Uganda. Nationally, 11% of women report that their first sexual encounter was forced [44].

In Uganda, radio is the most popular form of media, with a plethora of national, regional and local radio stations. The 2015 BBC World Service’s nationally representative survey found that the majority (87%) of the Ugandan adult population had a working radio, in contrast to only a third (34%) having a working TV [45]. Radio listenership is high in both urban and rural areas (80% in urban, 76% in rural), unlike television viewing which is much more heavily skewed towards urban populations [46]. For most households in Kasese District, as well as nationally, radio provides the main source of information and news.

### SASA! Together

**Fig. 1:**
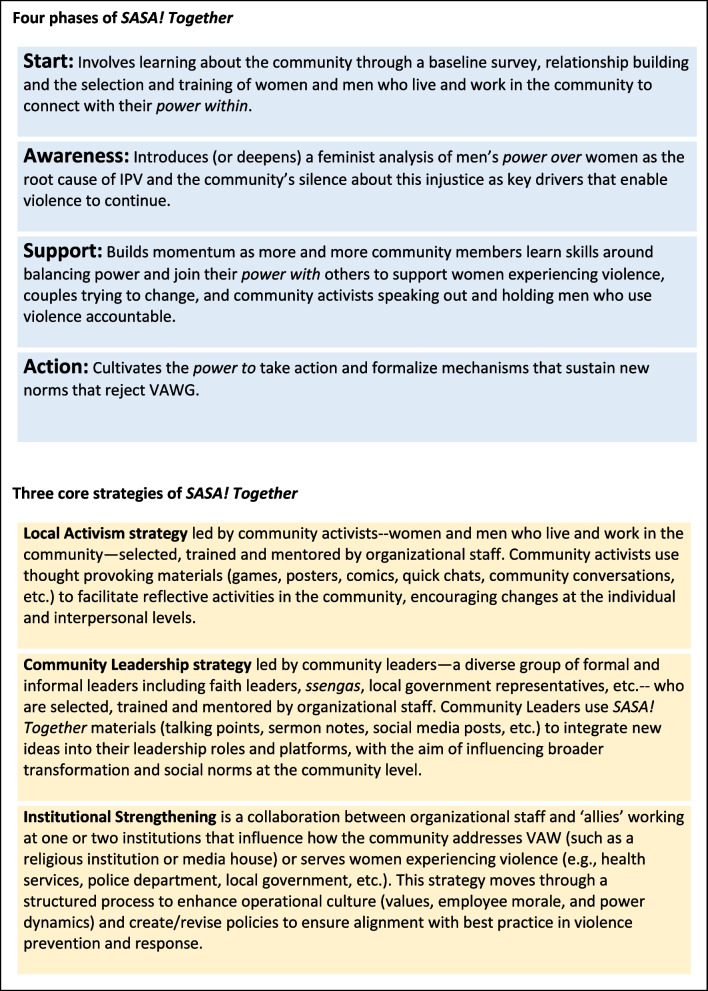
*SASA! Together* phases and core strategies

*SASA! Together* is the revised version of *SASA!*, an evidence based community mobilisation approach to prevent violence against women [47]. Both *SASA!* and *SASA! Together* aim to transform imbalances of power and change the underlying norms that perpetuate VAW by sparking community-wide critical discussion and positive action. The intervention seeks to shift community-wide gendered power imbalances, with a key focus on balancing power within intimate relationships. The approach supports whole communities through a phased process of change, with phases analogous to processes set out by Prochaska et. al (1992) in their individual-level behaviour-change Stages of Change Theory [48]. *SASA!* is both a Kiswahili word that means “now”, underscoring the urgent need to prevent VAW, and the acronym for its four phases – Start, Awareness, Support, Action (Fig. 1). *SASA! Together* is typically implemented over a three-to-four year period, and involves three core strategies, facilitated by different groups of community members: Local Activism; Community Leadership; and Institutional Strengthening (Fig. 1).

*SASA! Together* was designed by Raising Voices and, since 2019, has been implemented in Kasese by NGO Uganda Network on Law Ethics and HIV/AIDS (UGANET) (with technical support from Raising Voices). Raising Voices’ technical support includes training and mentorship for UGANET staff, who in turn cascade training to Community Activists (CAs), as described in more detail elsewhere (Hameed S, Musenze J, Awino E, Abramsky T, Witt A, Nakuti J, Buller A: Assessing Together with Gloria: A Process Evaluation of an edutainment-based intervention to prevent violence against women in Uganda, Submitted). Due to the rugged nature of the terrain, and different levels of programming intensity in different areas, implementation has not been uniform across the study area. While all villages were considered part of the *SASA! Together* programming area, and all initially had at least one designated CA within, some villages no longer had an active CA at the time the evaluation study commenced (the CA either having moved away or opted to no longer participate). Villages with inactive CAs were nevertheless still included in programming (albeit at a lower intensity), with activities conducted by visiting CAs from other villages. It should also be noted that a CA’s level of activity (active/inactive designation) should be considered fluid and may potentially change (more than once) over the course of *SASA! Together* implementation.

### The SASA! Together radio drama – ‘Together with Gloria!’

‘Together with Gloria!’ (TWG) comprises a series of 33 episodes, with themes and ideas around power and relationships that evolve over the course of the drama (aligned with other *SASA! Together* content). Because in-person *SASA! Together* programming was already underway and had a longer planned duration than TWG, TWG content was designed to deepen engagement with *SASA! Together* ideas, rather than run precisely parallel to community implementation of each phase. Broadcasting began during the Awareness phase of community-based in-person programming, and continued through the transition to the Support phase. TWG was conceptualised, developed and written in partnership by Peripheral Vision International (PVI), an NGO specialising in the use of media to catalyse social change, and Raising Voices, with input also provided from LSHTM and UGANET. TWG was written in English, and then translated and broadcast in Lukhonzo.

For this initial pilot, TWG was aired on three local radio stations in Kasese (from June 2022 to January 2023), the three stations selected to ensure coverage spanned different demographic and religious communities. New episodes (lasting an average of 21 min each) were aired on a weekly basis, with each radio station broadcasting the episode three times during the week (a total of 9 broadcasting time-slots per episode, spanning morning, afternoon and evening times).

In addition to the radio broadcasting, Community Activists (CAs), trained by UGANET in effective facilitation of conversations around power and violence, promoted TWG and were supported to convene and facilitate ‘Radio Discussion Groups’. These groups, usually piggy-backing onto pre-existing groups such as savings groups, comprised small numbers of community members who would meet on a weekly basis to listen to the latest episode and participate in guided discussion of its content. The aim of this guided discussion was to help listeners further deepen their engagement with the ideas and issues raised in the episode.

To facilitate this process, and enhance potentials for synergy, Raising Voices created a discussion guide for each episode to support partners (in this case UGANET) in implementing radio components (specifically Radio Discussion Groups) alongside in-person *SASA! Together* programming. They also provided technical support to UGANET during the implementation of the radio drama.

### Evaluation design

The evaluation was designed to assess the potential for the *SASA! Together* radio drama to strengthen the delivery and *potential* impacts of in-person *SASA! Together* community programming. It is a ‘proof of concept’ study rather than a full-scale impact evaluation, with the broad objectives of exploring (1) the extent to which the radio drama expands the reach of in-person *SASA! Together* programming; (2) the mechanisms through which the radio drama might ‘boost’ the effects of in-person *SASA! Together*; (3) community members’ perceptions of the radio drama’s influence; (4) change in attitudes relating to IPV/relationships, quality of intimate relationships, and activism/intention to prevent or respond to IPV. This paper focuses on the first of these objectives. Analyses relating to the other objectives, as well as a complementary process evaluation conducted with staff from all partner organisations, will be described elsewhere.

The evaluation study employed mixed methods: repeated cross-sectional surveys of community members; focus group discussions (FGD) and in-depth interviews (IDI) with community members, listener group members, *SASA! Together* CAs, and community leaders; and audio recordings of listener group discussions.

### Community-member surveys

The quantitative component of the study comprised two cross-sectional surveys of community members. The first (baseline) was conducted approximately 6 weeks after TWG began airing, and the second (endline) approximately 33 weeks later (~ 6 weeks after Radio programming finished). The survey questionnaire was designed specifically for this study, and at both time-points, the survey collected data on TWG reach/listenership, attendance/involvement in other *SASA! Together* activities, attitudes towards IPV and intimate relationships, quality of intimate relationships, and activism/intention to prevent or respond to IPV (see Additional File 1). A planned midline survey (~ 19 weeks after baseline) was cancelled due to the Ebola outbreak in September 2022.

#### Sampling strategy, sample size and eligibility criteria

At baseline we sampled 726 households with the aim of completing 650 interviews with community members (325 women and 325 men). At endline, we increased our sample size, using additional resources freed up by cancellation of the midline survey. We thus sampled 1080 households, aiming to complete 980 interviews (490 women and 490 men). This oversampling of households was done to allow for failure to contact respondents, refusals to participate, and lack of eligible respondents in selected households.

We employed a multi-stage sampling strategy, with village as the primary sampling unit (PSU). The survey was conducted in 18 villages in Kisinga Sub County (encompassing approximately 5560 households), all of which were covered by in-person *SASA! Together* programming (though levels of programming activity varied between villages – see above). This included 3 less accessible villages in order to estimate intervention reach to these more remote locations. We did not exclude villages with currently inactive CAs as progamming was still occurring within these villages (albeit to a less optimal intensity), and indeed a rationale behind TWG was to plug the gaps in reach left by in-person programming and its associated implementation challenges.

From these 18 villages we used stratified random sampling (by urban/rural, active/inactive CA and estimated population size) to select 9 villages to form the basis of our female sample. The remaining 9 formed the basis of our male sample. The designation of male/female villages remained the same for the baseline and endline surveys to maintain comparability between survey rounds.

Within each village, 40 households were sampled at baseline, and 60 at endline. The sampling strategy was explicitly designed to avoid resampling the same households in multiple survey rounds. Households were selected using systematic sampling based on the ‘random walk’ procedure from a central point in the village (identified with the help of local officials), with direction of travel established by spinning a bottle, and sampling interval pre-determined as 3. Selection of households for baseline, and listing of households (using GPS coordinates) for endline, were undertaken during the same ‘random walk’, with endline households listed from within the baseline sampling interval to ensure that both samples were drawn from the same neighbourhoods. No substitutions were made where enumerators were unable to make contact with a selected household (after three attempts) or where households refused to participate.

One eligible person was randomly selected from each sampled household to participate in the survey. A limit of one respondent per household was set to ensure respondent safety and confidentiality. A person was eligible for inclusion in the survey if they were aged 18 years or above, usually resident in the household, were proficient in the Lukhonzo language, and were the same sex as their village sampling designation (e.g. only women were eligible in ‘female’ villages).

Details of consent procedures, survey conduct and ethical approval are provided after the qualitative methods.

#### Statistical analysis

Survey responses were entered directly on to a tablet using ODK software with automatic encryption. Data were analysed in Stata SE.

We produced descriptive data for each time-point, disaggregated by sex, for indicators relating to TWG’s reach and exposure to in-person *SASA! Together* programming. In addition to producing overall proportions, we also present the median, interquartile range (IQR) and range of village-level exposure data to explore geographical variation in reach. Overlap between exposure to TWG and exposure to in-person *SASA! Together* programming was explored descriptively.

Chi-squared tests of association and logistic regression with cluster robust standard errors (to account for the clustered nature of the data) were used to assess differences in TWG’s reach between different sub-groups of the population (by village-level characteristics, respondent age, education, current relationship status, number of dependent children, disability status, religion, radio ownership and usual frequency of radio listening). Odds ratios of association between each of these characteristics and ever having listened to TWG, adjusted for radio ownership, were also estimated to explore the extent to which observed associations were explained by differences in radio ownership between different sub-groups.

We also present comparable data on exposure to in-person *SASA! Together* programming broken down by demographic characteristics, to allow a descriptive comparison between levels of exposure to in-person programming and levels of exposure to TWG.

Descriptive data on shared listening and diffusion, as well as barriers to listening, were disaggregated by sex and survey round.

### Qualitative methods

The qualitative component of the evaluation comprised in-depth interviews (IDIs) and focus group discussions (FGDs). The various time-points at which each method was used are presented in Additional File 2.

#### In-depth interviews (IDIs) with listeners

IDIs were conducted with community members in Kisinga Sub County who, at the time of first interview, were identified as “listeners” to TWG.

We conducted a total of 44 IDIs with listeners, 24 at midline (19 weeks into broadcasting, conducted by telephone due to the Ebola outbreak), and 20 at endline (in-person). Eighteen people were interviewed at both midline and endline, thus comprising our listeners ‘cohort’, allowing us to explore how experiences of and engagement with TWG changed over time.

Listener interviewees were recruited from *SASA! Together* Radio Discussion Groups, to ensure that they had at least some exposure to TWG, and purposively selected to represent different frequencies of listenership. CAs identified and (with their consent) shared phone numbers of potential participants with UGANET. These contact details were then passed on to the data collectors, Research World International (RWI), for the purpose of recruiting participants.

Interviewers telephoned potential participants to explain the study and ask their consent to participate in an IDI. If consent was obtained, the interviewer either conducted the IDI then or arranged a future time to do so.

#### IDIs with community activists

Twenty-seven IDIs were conducted with Community Activists (CAs): 15 were interviewed at midline (by telephone), 12 of whom were interviewed again at endline (in person). Thus, longitudinal data is available from 12 CAs.

To recruit participants, members of staff from UGANET obtained potential participants’ phone numbers, and shared these with RWI for the recruitment process. Interviewers then followed the same recruitment/interview process as for the listener IDIs.

#### Focus group discussions (FGDs)

At baseline, four FGDs were conducted—two with Listener Groups (one female group, one male group), and two with CAs (one female group, one male group). Endline FGDs were conducted with: two Listener Groups (one female group, one male group); two groups of CAs (one female group, one male group); and one group of Community Leaders (cultural/religious/local government). Listener group and CA FGDs were conducted in single-sex groups to facilitate equal participation by each sex (some women in this setting might lack confidence and/or assertiveness in the presence of men) and to encourage frank expression of opinions and experiences.

Radio Discussion Groups were identified and selected with the help of *SASA! Together* CAs who had been involved in setting up and facilitating these groups. CA FGD participants were purposively selected by UGANET to represent a range of ages, duration of involvement with the intervention and village-level characteristics (e.g. urban/rural). UGANET also helped identify community leaders (cultural, religious and local government) with whom they had had involvement during implementation of *SASA! Together* programming.

Recruitment of individuals for focus groups was led by RWI in order to maintain the independence of the research process.

#### Qualitative data management and analysis

All IDIs and FGDs were audio recorded with the participants’ consent. Audio recordings and hand-written notes were transcribed verbatim and translated from Lukhonzo into English. A sample of the transcripts were checked by an independent research consultant for quality of transcription and translation. Data were anonymised and uploaded onto NVivo and analysed following a qualitative thematic analysis approach in order to meet the objectives of the study.

Personal contact information for IDI and FGD participants was encrypted and stored in a separate password-protected server location to the transcripts, and will be destroyed within 12 months of publication of study reports. Consent forms were stored securely in locked filing cabinets in secure locations arranged by RWI, and photos of the forms uploaded onto a secure server. The paper forms were transferred to locked filing cabinets in Raising Voices offices upon completion of each round.

#### Ethical considerations, informed consent and research procedure

The survey, IDIs and FGDs were carried out by Research World International Limited (RWI), though fieldwork protocols and the training of enumerators was overseen by LSHTM and Raising Voices staff.

Individual written informed consent was obtained from all individuals participating in the survey, and in-person IDIs and FGDs. In the case of telephone interviews, interviewers sought verbal consent for participation. If the participant gave verbal consent, the interviewer attested to this by writing the name of the participant on a paper copy of the consent form and signing and dating their own name on the same form.

The research was conducted in accordance with international guidelines for the collection of data on VAW [49]. These guidelines seek to minimise reporting bias and risk of harm to respondents and interviewers. Interviewers, all fluent in the main local language, Lukhonzo, underwent 3 weeks of preparatory training on ethical and methodological issues surrounding the conduct of research relating to IPV. Survey interviews and in-person IDIs were conducted in Lukhonzo in a private place of the respondent's choosing, and conducted by interviewers that were the same sex as the respondent. On conclusion of the interview, respondents were offered a card containing information on support services in the area, with referral protocols enacted (via UGANET) in cases where women disclosed recent experiences of violence, reported feeling unsafe or at risk of imminent violence, or reported violence towards a child. Locations of FGDs were selected by the field coordinator with the help of CAs, to ensure privacy and safety of participants.

The study received ethical approval from the Institutional Review Boards at the London School of Hygiene and Tropical Medicine (UK), Mildmay Uganda, and the Uganda National Council for Science and Technology. Approval to work in the study communities was obtained from local government offices and Community leaders.

## Results

### Response rates and sample characteristics

Six of the eighteen study villages were classed as urban (three female, three male) and the rest as rural, with one male village and two female villages at least a three to four hour walk from the next nearest village (‘less accessible’). Twelve of the villages (six female, six male) had an active *SASA! Together* CA.

At baseline, we completed interviews with 298 men and 346 women (98% response rate among households in which an eligible member was identified). At endline, we interviewed 512 men and 506 women (> 99% response rate).

Respondent characteristics for the two surveys are presented in Table 1. At baseline, the age of respondents ranged from 18 to 90 years old, with men, on average, slightly older than women (median age of 38 versus 32 years). Most respondents had primary education only, with men more likely than women to have above primary-level education (baseline—35% versus 28%). Three-quarters of the baseline sample were married/cohabiting, and male respondents were more likely than female respondents to be childless (58% versus 30%). The majority of respondents were Anglican or Catholic, and all reported that Lukhonzo was their main language (proficiency in Lukhonzo was one of the eligibility criteria).

**Table 1:** Sample characteristics at Baseline and Endline, disaggregated by sex

	Baseline		Endline		Baseline/Endline comparison χ^2^ *p*-value	
	Male *N* = 298	Female *N* = 346	Male *N* = 512	Female *N* = 506	Male sample	Female sample
	n (%)	n (%)	n (%)	n (%)		
Demographics						
Age (years)						
* 18–25*	76 (25.5%)	105 (30.3%)	176 (34.4%)	128 (25.3%)		
* 26–35*	58 (19.5%)	100 (28.9%)	144 (28.1%)	160 (31.6%)		
* 36–50*	91 (30.5%)	86 (24.9%)	132 (25.8%)	160 (31.6%)		
* 51–90*	73 (24.5%)	55 (15.9%)	60 (11.7%)	58 (11.5%)		
	χ^2^ *p*-value = 0.002		χ^2^ *p*-value = 0.011		< 0.001	0.033
Highest level of education						
* Primary or none*	195 (65.4%)	250 (72.3%)	289 (56.6%)	391 (77.3%)		
* Secondary or higher*	103 (34.6%)	96 (27.7%)	222 (43.4%)	115 (22.7%)		
	χ^2^ *p*-value = 0.062		χ^2^ *p*-value < 0.001		0.013	0.096
Current relationship status						
* Married/cohabiting*	234 (78.5%)	251 (72.5%)	331 (64.6%)	381 (75.3%)		
* Regular partner living apart*	10 (3.4%)	2 (0.6%)	11 (2.1%)	4 (0.8%)		
* No regular partner*	54 (18.1%)	93 (26.9%)	170 (33.2%)	121 (23.9%)		
	χ^2^ *p*-value = 0.002		χ^2^ *p*-value < 0.001		< 0.001	0.590
Number of children < 18 years that respondent is responsible for						
* None*	174 (58.4%)	105 (30.3%)	193 (37.7%)	204 (40.3%)		
* 1–3*	87 (29.2%)	132 (38.2%)	209 (40.8%)	218 (43.1%)		
* 4* +	37 (12.4%)	109 (31.5%)	110 (21.5%)	84 (16.6%)		
	χ^2^ *p*-value < 0.001		χ^2^ *p*-value = 0.140		< 0.001	< 0.001
Religion						
* Catholic*	102 (34.2%)	156 (45.1%)	196 (38.3%)	242 (47.8%)		
* Anglican*	171 (57.4%)	148 (42.8%)	245 (47.9%)	210 (41.5%)		
* Other*	25 (8.4%)	42 (12.1%)	71 (13.9%)	54 (10.7%)		
	χ^2^ *p*-value = 0.001		χ^2^ *p*-value = 0.007		0.011	0.670
Lukhonzo is main language spoken at home						
* Yes*	298 (100.0%)	346 (100.0%)	512 (100.0%)	506 (100.0%)		
Sources of media access						
Household owns a working radio	187 (62.8%)	154 (44.5%)	294 (57.4%)	234 (46.3%)		
	χ^2^ *p*-value < 0.001		χ^2^ *p*-value < 0.001		0.140	0.600
Someone in household owns a mobile phone						
* Any smart phone*	49 (16.4%)	42 (12.2%)	75 (14.7%)	29 (5.7%)		
* Only regular mobile(s)*	167 (56.0%)	223 (64.6%)	231 (45.2%)	256 (50.6%)		
* No mobile*	82 (27.5%)	80 (23.2%)	205 (40.1%)	221 (43.7%)		
	χ^2^ *p*-value = 0.074		χ^2^ *p*-value < 0.001		0.001	< 0.001
How often listen to radio in past 3 months						
* Never*	44 (15.0%)	121 (35.5%)	69 (13.6%)	170 (34.2%)		
* Less than once per week*	27 (9.2%)	40 (11.7%)	100 (19.7%)	115 (23.1%)		
* At least once per week*	86 (29.3%)	67 (19.6%)	111 (21.9%)	97 (19.5%)		
* Most days*	137 (46.6%)	113 (33.1%)	227 (44.8%)	115 (23.1%)		
	χ^2^ *p*-value < 0.001		χ^2^ *p*-value < 0.001		< 0.001	< 0.001
Usually listen on a traditional radio or mobile phone? (if ever listen to radio)						
* Radio (battery operated)*	205 (81.3%)	135 (60.3%)	272 (62.0%)	239 (71.6%)		
* Radio (mains or solar)*	13 (5.2%)	55 (24.6%)	111 (25.3%)	45 (13.5%)		
* Mobile phone*	19 (7.5%)	27 (12.1%)	44 (10.0%)	49 (14.7%)		
* Radio and mobile phone*	15 (6.0%)	7 (3.1%)	12 (2.7%)	1 (0.3%)		
	χ^2^ *p*-value < 0.001		χ^2^ *p*-value < 0.001		< 0.001	< 0.001
Used other sources to access media and information in past 3 months						
* Television*	132 (44.3%)	36 (10.4%)	245 (47.9%)	27 (5.3%)		
	χ^2^ *p*-value < 0.001		χ^2^ *p*-value < 0.001		0.330	0.005
* Newspapers/magazines*	55 (18.5%)	2 (0.6%)	94 (18.4%)	62 (12.3%)		
	χ^2^ *p*-value < 0.001		χ^2^ *p*-value = 0.007		0.970	< 0.001
* Social media*	41 (13.8%)	19 (5.5%)	86 (16.8%)	23 (4.5%)		
	χ^2^ *p*-value < 0.001		χ^2^ *p*-value < 0.001		0.250	0.530
* Online news sites/other websites*	4 (1.3%)	4 (1.2%)	31 (6.1%)	7 (1.4%)		
	χ^2^ *p*-value = 0.830		χ^2^ *p*-value < 0.001		0.001	0.770

Household radio ownership was higher among men than women (baseline—63% versus 45%), as was regularity of listening to the radio. Men were more likely than women to report listening to the radio most days (47% versus 33%), and less likely to report never listening (15% versus 36%). Most people reported listening to the radio on traditional radios rather than mobiles phones, with smart phone ownership relatively low overall (baseline – 16% of men, 12% of women). Use of television to access information (past 3 months) was less common than radio, and exhibited an even starker gender difference (baseline – 44% of men versus 10% of women). Use of newspapers/magazines, social media and other online sources of news was much lower and also predominantly reported by male respondents.

Some differences were noted between our baseline and endline samples. Men in the endline sample were younger, more highly educated and less likely to have a regular partner than men in the baseline sample. At endline, men were less likely to be childless than their baseline counterparts, while women were more likely to be childless than their baseline counterparts.

### Overall reach of Together with Gloria!

At baseline, men were more likely than women to have heard of TWG (55% versus 47%) and to have ever listened to TWG (50% versus 33%) (Table 2). Reported listenership was higher at endline among both men (53%) and women (51%), with the baseline sex-difference eliminated by a particularly marked increase in listening among women. Among ever listeners at endline, over half (60% of men and 56% of women) listened most or every week, and just over 70% (men and women) had listened to episodes since Christmas 2022 (the final 5 weeks of TWG broadcasting).

**Table 2:** Overall exposure to TwG and in-person *SASA! Together* community programming

	Baseline			Endline			Baseline/Endline comparison	
	Men	Women	χ^2^ *p*-value	Men	Women	χ^2^ *p*-value	Men’s sample	Women’s sample
	*N* = 298	*N* = 346		*N* = 512	*N* = 506			
Ever heard of TwG	165 (55.4%)	164 (47.4%)	0.044	352 (68.9%)	319 (63.0%)	0.049	< 0.001	< 0.001
Ever listened to TwG	148 (49.8%)	115 (33.2%)	< 0.001	268 (52.5%)	260 (51.4%)	0.71	0.46	< 0.001
Listened in final 5 weeks of airing	-	-	-	190 (37.3%) *(70.9% of ever listeners)*	186 (36.8%) *(71.5% of ever listeners)*	0.85	-	-
Listened most/every week(s)	-	-	-	160 (31.6%) *(59.7% of ever listeners)*	146 (30.2%) *(56.2% of ever listeners)*	0.64	-	-
Any exposure to in-person *SASA! Together* programming	67 (22.5%)	18 (5.2%)	< 0.001	77 (15%)	81 (16.0%)	0.67	0.008	< 0.001

**Fig. 2:**
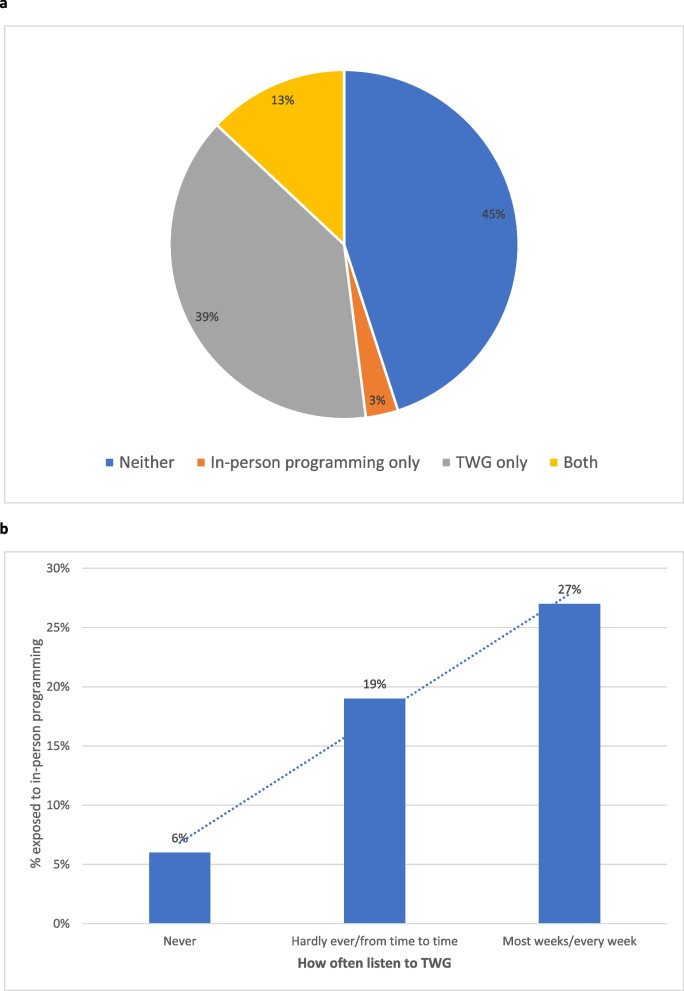
**a** Exposure to TWG and in-person *SASA! Together* programming (among men and women) (Endline, *n* = 1,016). **b** Exposure to in-person *SASA! Together* programming (men and women), by TWG listening frequency (Endline, *n* = 989)

Reported exposure to in-person *SASA! Together* programming was substantially lower than exposure to TWG, at both baseline and endline (Table 2). As with TWG exposure, in-person *SASA! Together* exposure was lower among women than men at baseline, but no gender disparity was observed at endline (endline exposure—15% of men, 16% of women). While most people who had been exposed to in-person *SASA! Together* programming had also been exposed to TWG (13% of the total sample at endline had been exposed to both, versus 3% to *SASA! Together* only), TWG reached many who had no exposure to in-person *SASA! Together* programming (39% of the sample) (Fig. 2a). There was evidence of an association between the two exposures; TWG listeners were more likely than non-listeners to have been exposed to in-person *SASA! Together* programming, with likelihood of exposure increasing the more often respondents listened to TWG (Fig. 2b).

**Fig. 3:**
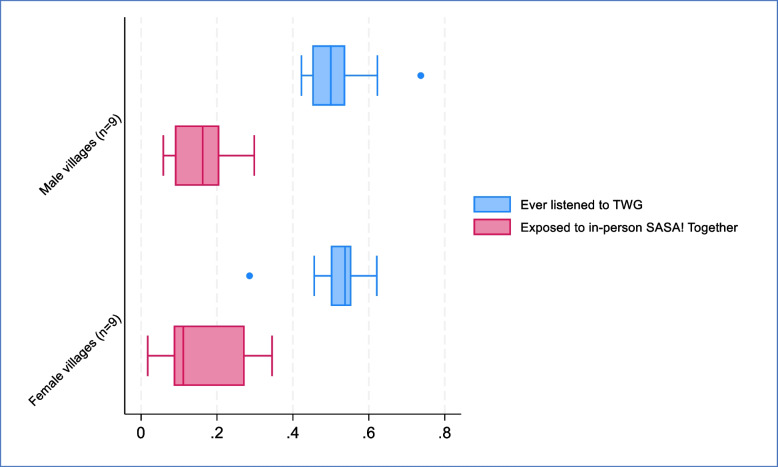
Village-level estimates of exposure to TWG and in-person *SASA! Together* (Endline)

Endline village-level variation in exposure to TWG and *SASA! Together* community programming is presented in Fig. 3. Within each village, exposure to TWG was higher than exposure to in-person *SASA! Together* programming, and, across all villages but one, exposure to TWG was higher than the maximum village-level estimate for exposure to in-person *SASA! Together* community programming (Fig. 2). There was also higher inter-village variation in exposure to in-person *SASA! Together* programming than there was for exposure to TWG. At endline, the proportion with exposure to TWG ranged from 0.42 to 0.74 for men (IQR 0.45–0.54; ICC 0.024), and 0.29 to 0.62 for women (IQR 0.5 to 0.55; ICC 0.023). Respective figures for in-person *SASA! Together* programming were 0.06 to 0.30 for men (IQR 0.09–0.21; ICC 0.051) and 0.02 to 0.34 for women (IQR 0.09–0.27; ICC 0.169).

There were no consistent patterns of association between village-level characteristics and exposure to TWG, with no significant differences observed between urban and rural villages, nor those with an active versus no/inactive CA (Table 3). While there was some suggestion that men in the less accessible villages were slightly less likely to listen than men in the more accessible villages, the opposite trend was observed among women. A similar lack of consistent pattern was observed in relation to exposure to in-person *SASA! Together* programming; women in urban villages were less likely to have exposure than those in rural villages, while men in less accessible villages were less likely to have exposure than those in more accessible villages (Supplementary Table 2).

**Table 3:** Characteristics associated with ever having listened to TwG (Endline)

	Men (*N* = 512)					Women (*N* = 506)				
				OR (95%CI) for ever having listened to TwG					OR (95%CI) for ever having listened to TwG	
	N	Own a radio	Ever listened to TwG	Crude OR	OR adjusted for radio ownership	N	Own a radio	Ever listened to TwG	Crude OR	OR adjusted for radio ownership
Village-level characteristics										
Rural/Urban										
* Rural*	354	201 (57%)	193 (55%)	-	-	334	158 (47%)	183 (55%)	-	-
* Urban*	158	93 (59%)	75 (48%)	0.76 (0.50–1.14)	0.73 (0.45–1.17)	172	76 (44%)	77 (45%)	0.67 (0.37–1.21)	0.65 (0.33–1.29)
Village has a *SASA! Together* CA?										
* No CA/inactive*	141	73 (52%)	66 (47%)	-	-	169	82 (49%)	94 (56%)	-	-
* Active CA*	371	221 (60%)	202 (55%)	1.35 (0.95–1.92)	1.26 (0.84–1.88)	337	152 (45%)	166 (49%)	0.77 (0.50–1.19)	0.79 (0.40–1.55)
Distance from other villages										
< *3 h walk from other villages*	461	272 (59%)	245 (53%)	-	-	397	196 (49%)	197 (50%)	-	-
* 3–4 h walk from other villages*	51	22 (43%)	23 (45%)	0.72 (0.55–0.94)	0.84 (0.62–1.14)	109	38 (35%)	63 (58%)	1.39 (0.94–2.06)	2.04 (1.24–3.37)
Individual-level characteristics										
Age-group										
* 18–25 years*	176	105 (60%)	77 (44%)	-	-	128	55 (43%)	68 (53%)	-	-
* 26–* *35 years*	144	82 (57%)	77 (54%)	1.5 (0.98–2.28)	1.61 (0.98–2.65)	160	80 (50%)	80 (50%)	0.88 (0.53–1.48)	0.74 (0.44–1.25)
* 36–50 years*	132	73 (55%)	74 (56%)	1.67 (1.06–2.63)	1.84 (1.08–3.14)	160	71 (45%)	79 (49%)	0.86 (0.41–1.80)	0.82 (0.41–1.64)
* 51–90 years*	60	34 (57%)	40 (67%)	2.57 (1.45–4.55)	2.88 (1.50–5.54)	58	28 (48%)	33 (57%)	1.16 (0.51–2.65)	1.08 (0.51–2.28)
				*1.33 (1.20–1.47)*	*1.39 (1.23–1.56)*				*1.01 (0.77–1.33)*	*1.00 (0.77–1.30)*
Education										
* Primary or none*	289	153 (53%)	150 (52%)	-	-	391	170 (44%)	188 (48%)	-	-
* Secondary or higher*	222	141 (64%)	118 (54%)	1.07 (0.73–1.58)	0.96 (0.65–1.41)	115	64 (56%)	72 (63%)	1.81 (1.21–2.70)	1.59 (1.07–2.36)
Current relationship status										
* Regular partner*	342	200 (58%)	193 (57%)	-	-	385	181 (47%)	205 (53%)	-	-
* No regular partner*	170	94 (55%)	94 (44%)	0.60 (0.41–0.89)	0.60 (0.37–0.96)	121	53 (44%)	55 (45%)	0.73 (0.47–1.13)	0.73 (0.47–1.13)
Number of children < 18 years that respondent is responsible for										
* 0*	193	117 (61%)	90 (47%)	-	-	204	96 (47%)	125 (61%)	-	-
* 1–3*	209	119 (57%)	112 (54%)	1.30 (0.95–1.77)	1.38 (0.92–2.07)	218	96 (44%)	92 (42%)	0.46 (0.31–0.69)	0.41 (0.25–0.66)
* 4* +	110	58 (53%)	66 (60%)	1.68 (1.01–2.80)	1.92 (1.02–3.63)	84	42 (50%)	43 (51%)	0.66 (0.34–1.28)	0.56 (0.31–1.00)
				*1.30 (1.02–1.65)*	*1.39 (1.01–1.89)*				*0.73 (0.54–0.99)*	*0.67 (0.50–0.89)*
Have a physical disability (excluding hearing loss)										
* No*	473	276 (58%)	244 (52%)	-	-	478	223 (47%)	247 (52%)	-	-
* Yes*	39	18 (46%)	24 (62%)	1.49 (0.73–3.03)	1.77 (0.87–3.62)	28	11 (39%)	13 (46%)	0.81 (0.43–1.52)	0.90 (0.47–1.73)
Have some difficulty hearing										
* No*	487	285 (59%)	259 (53%)	-	-	487	222 (46%)	252 (52%)	-	-
* Yes*	25	9 (36%)	9 (36%)	0.49 (0.24–1.00)	0.60 (0.31–1.15)	19	12 (63%)	8 (42%)	0.68 (0.32–1.43)	0.43 (0.21–0.86)
Religion										
* Catholic*	196	122 (62%)	96 (49%)	-	-	242	109 (45%)	121 (50%)	-	-
* Anglican*	245	131 (53%)	132 (54%)	1.19 (0.83–1.72)	1.34 (0.89–2.01)	210	97 (46%)	114 (54%)	1.19 (0.74–1.90)	1.20 (0.70–2.07)
* Other*	71	41 (58%)	40 (56%)	1.32 (0.80–2.17)	1.41 (0.81–2.46)	54	28 (53%)	25 (46%)	0.86 (0.33–2.27)	0.74 (0.28–1.97)
Own a radio										
* No*	218	-	81 (37%)	-	-	271	-	86 (32%)	-	-
* Yes*	294	-	187 (64%)	3.01 (1.75–5.19)	n/a	234	-	174 (74%)	6.24 (4.50–8.66)	n/a
Radio listening frequency										
* Less often than most days*	280	-	105 (38%)	-	-	382	-	171 (45%)	-	-
* Most days*	227	-	162 (72%)	4.19 (2.57–6.84)	3.27 (2.16–4.95)	115	-	87 (76%)	3.83 (2.27–6.48)	1.94 (1.17–3.21)

### Who listened to Together with Gloria?

Table 3 presents endline data on associations between respondent characteristics and ever having listened to TWG.

#### Women

Women across all age-groups were equally likely to have ever listened to TWG. However, listening patterns varied with respect to other characteristics. Women with secondary education (or above) were more likely to listen than women with primary only/no education (63% versus 48%). This is in part, though not wholly, explained by their increased likelihood of living in a household with a radio. They were less likely to listen if they had children (compared to those with none), but no trend was observed in relation to increasing number of children. We did not find evidence of an association between disability (either non-hearing related or impaired hearing) and listening, though small numbers reporting disability limited our power to explore these associations. Nor did we observe any difference in listening levels between women belonging to different religions. Women were much more likely to listen to TWG if someone in their household owned a radio, compared to those with no radio in the household (74% versus 32%) and if they reported that they usually listened to the radio (not TWG) most days/every day, compared to if they usually listened less often.

#### Men

Among men, probability of listening increased with age (67% among > 50 yr olds versus 44% in 18–25 yr age-group). Those in a current relationship were more likely to listen than those with no regular partner (57% versus 44%), as were those with a greater number of children compared to those with none/fewer (60% if 4 + children; 54% if 1–3 children; 47% if no children). There was no association between listening and having a non-hearing related disability, but weak evidence that those with a hearing impairment were less likely to listen than those without (36% versus 53%). Those with a hearing impairment were also less likely to live in a household in which someone owned a radio. No association was observed with education or religion. As with women, TWG listening was higher among men living in households with radios (compared to no radio) (64% versus 37%) and among those who reported regularly listening to the radio (compared to those who listened less often).

#### Within sub-group comparisons between TWG listening and exposure to in-person SASA! Together programming

In every sub-group of women and men, including those with lower relative odds of TWG listening, exposure to TWG was higher than exposure to in-person *SASA! Together programming* (see Additional File 3).

### Shared listening and diffusion

**Fig. 4:**
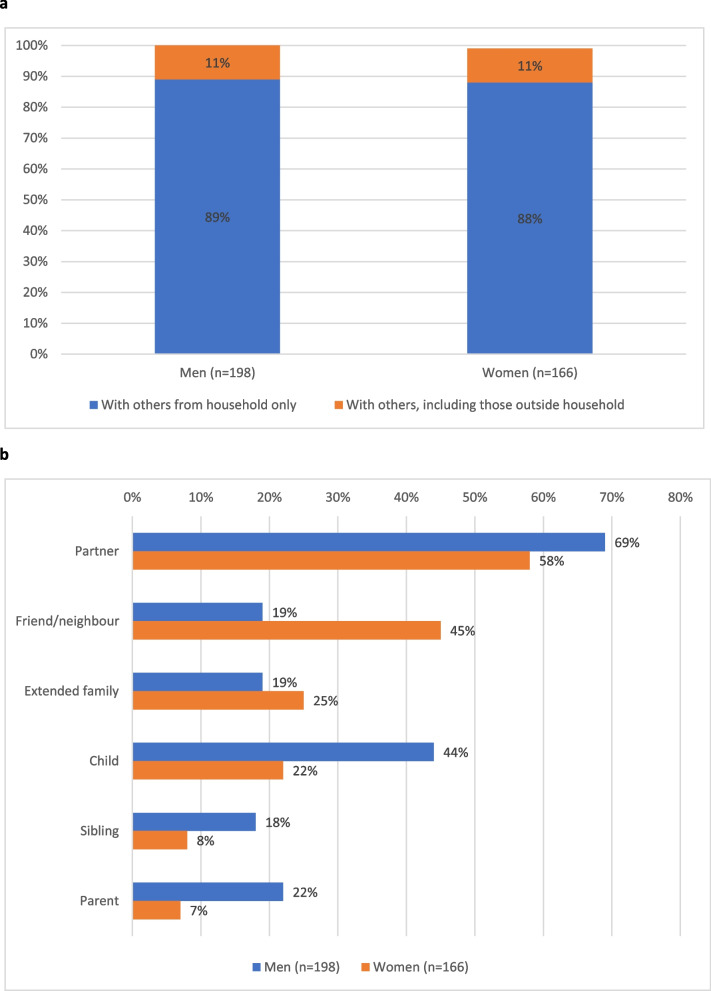
**a** Who usually listen to TWG with, among those who report usually listening with others (Endline). **b** People respondent has listened to TWG with (ever), among those usually listening with others (Endline)

A high proportion of TWG listeners reported ‘usually’ listening to TWG with others (endline—74% of male listeners; 64% of female listeners). Among those usually listening with others, the vast majority (almost 90%) reported listening with people from their own households, while approximately 10% reported usually listening with others outside their households (Fig. 4a). More specifically, when asked who they had listened with (on any occasion), the most common response for both women and men was their partner (69% of men; 58% of women) (Fig. 4b). Additionally, a high percentage of women had listened with friends/neighbours (19% of men; 45% of women) and a high percentage of men had listened with children (44% of men; 22% of women). Listening also occurred (though to a lesser extent) with family of origin and other extended family members.

Listeners also reported discussing TWG with others in their community (not within Radio Discussion Groups). Among ever listeners at baseline, men were more likely than women to have discussed TWG with others (34% of male listeners, 14% of female listeners) (Fig. 4b). Discussion increased over the study period, particularly among women; in the endline survey, 41% of both male and female listeners reported discussing TWG with others in their community.

### Barriers and facilitators to listening

#### Barriers to listening – Findings from the quantitative survey

**Fig. 5:**
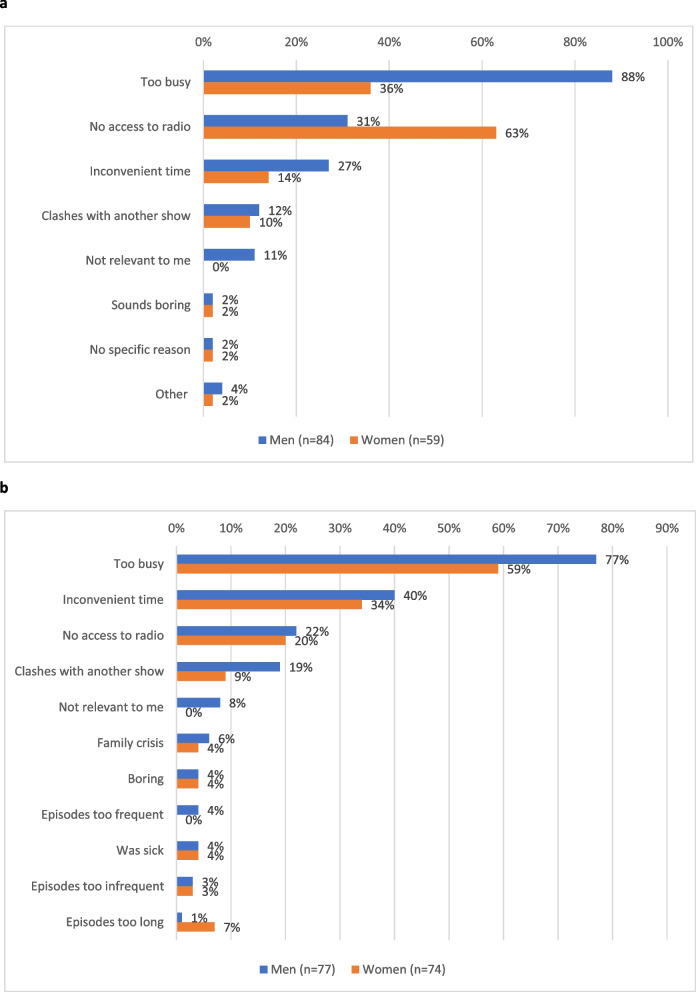
**a** Reasons never listened to TWG, among never-listeners who have heard of TWG (Endline). **b** Reasons not listened to TWG in recent weeks (since Christmas 2022), among ever-listeners (Endline)

The quantitative survey included two questions on reasons for not listening to TWG, the first asked to respondents who reported never having listened to TWG, and the second to respondents who had listened in the past but not recently (at endline ever-listeners were asked if they had listened since Christmas [final 5 weeks of broadcasting]). The endline responses are presented in Fig. 5a and 5b.

Reasons for never having listened differed somewhat between men and women (Fig. 5a). The most commonly cited reason for men was being too busy (88% of men; 36% of women), while women were most likely to cite lack of access to a radio (63% of women; 31% of men). These were also commonly cited reasons for having stopped listening in recent weeks. Both men and women were most likely to cite being too busy as the reason for no longer listening, followed by TWG being on at an inconvenient time. Lack of access to a radio was given as a reason for no longer listening by 22% of men and 20% of women. Very few respondents cited reasons related to the content or format of TWG, such as TWG being boring or episodes being too frequent or too long. In response to both questions, only men reported ‘lack of relevance to me’ as a reason for not listening (11% among those never listening, 8% among those who ceased listening) Fig. 5.

#### Barriers to listening – Qualitative findings

The qualitative findings were in line with the quantitative findings. Not having a radio was a key barrier, which was sometimes overcome by listening with neighbours and peers.

*“…it can be difficult because some of us we don’t have the radios then you run to the neighbour. Unfortunately, you find when she’s not there, then that can be the reason as to why you cannot manage to listen to it” (FGD with female listeners, Baseline)*

In addition, the qualitative data indicated issues with language and translation of the English script to Lukhonzo, the dominant language (among several other languages) spoken in Kasese. These reported issues included accent and dialect inaccuracies and use of non-colloquial words that led to dissatisfaction among listeners, in turn lessening their interest in the radio programme.

*“No, there are some people who say that there are some words… you find someone coming to ask you that I heard them mention the word ‘omwoleko’ and you wonder what the word ‘omwoleko’ means. Now […] for some of us, if you do not have grandparents who speak such words you can’t understand. This young generation of ours can’t understand what ‘omwoleko’ means.” (Interview with female listener, Midline)*

In many instances, the barriers reported by listeners could be linked to different characteristics and roles in society. For example, many of the young people interviewed described barriers such as missing the aired episodes because they were in school or socialising, or because they could not afford radios or batteries. Similarly, many male listeners reported that they could not regularly tune in because it conflicted with their work hours.

*“… [Male listeners] had a concern that not all people are always at home at that hour of the airing of the drama because others are businesspeople. Therefore, even the time that was allocated to the drama isn’t convenient because at times, someone may say ‘let me rush home and I go listen in’ but by the time they get home, they find when the drama has ended.” (Interview with Community Activist, Midline)*

Most female listeners, although motivated to listen to TWG, experienced barriers typical to their gendered roles in the community. Their barriers included having limited ability to decide what the family listened to on the radio, limited access to money to buy radios or batteries, and limited control over other duties and responsibilities in the household.

*“…at times it is hard for me because at the time of listening to the drama, your husband might tell you to do something else and you might not be able to listen in because of the work you have been told to do.” (FGD with female listeners, Baseline)*

#### Facilitators to listening – Qualitative findings

The qualitative data indicated that the availability of TWG on multiple radio stations at multiple times during the day were strong facilitators to listening. This helped address challenges of timing conflicting with work, and poor network coverage, as noted in this quote below.

*“Our Messiah Radio has one problem. Sometimes you tune in and it keeps losing network. But Musondolya is very clear, for it you can tune in and fine when its moving smoothly. But Messiah [Radio] keeps [being] unclear and the network keeps breaking…” (Interview with female listener, Midline)*

Indeed, the endline quantitative data indicated that 75% of male listeners and 54% of female listeners had listened to TWG on more than one radio station.

Other facilitators included a strong diffusion and peer effect. People listening together and discussing TWG with other community members contributed to intervention reach by helping recruit new listeners, and motivating and reminding people to keep listening.

*“My very first time hearing about it, I had just got off work from here in town. When I had just reached the backyard, I heard that there is a drama that is being played on the radio but it ends soon. That every morning at ten you will find it there, and in the evening at seven thirty you can find it there […]I said okay I will also listen to it. So the next day my neighbour came running for me ‘you come and listen to the drama that we are always telling you about every day…’” (IDI, Female listener, Midline)*

The female listeners in particular played a key role in motivating others in the community to tune in and listen, expanding TWG’s reach even further. In some instances, shared listening also provided a means of listening to those without radios in their own households.

Synergies were also observed between the above noted peer-effect and efforts by SASA*! Together* CAs to generate interest in TWG.

*“You know, at home, some used not listen to the drama. We were few who used to listen. Then with time, they also started listening to it because the SASA! people also continued to come to the community. And whenever they would come to play it in the community, I would call the neighbours and tell them that we go attend. They got involved in listening to the drama and now they are interested in listening to it the more. So, I see that I have motivated others.” (Interview with female listener, Endline)*

## Discussion

‘Together with Gloria!’ was widely listened to in our study communities. By the time the show had finished broadcasting, just over half of our sample reported having listened to TWG and, of these, 58% reported listening most or every week(s). Although women were less likely to have listened in the first few weeks of broadcasting, this gender disparity had disappeared by the time of our endline survey. Among men, listening was positively associated with older age, having a regular partner and having children, while women were more likely to listen if they had secondary education and were childless. Radio ownership was strongly associated with listening among both men and women. Overall, and within each demographic sub-group, exposure to TWG was much higher than exposure to in-person *SASA! Together* programming. TWG exposure was also more uniformly spread across study villages, evidence that TWG expanded the reach of *SASA! Together* both in terms of overall numbers and in terms of reducing geographical disparities in access. Both quantitative and qualitative findings suggest that people listened to, and spontaneously discussed, TWG with others in their households and communities. This communal listening, alongside the use of multiple broadcast stations/time-slots, were identified as factors facilitating listening. They helped to mitigate some of the common barriers to listening which included lack of radio access (particularly among women) and lack of time (particularly among men).

The ~ 50% reach of TWG compares favourably with other edutainment interventions around VAW and HIV prevention. The MTV Shuga television series, Down South 2, which focused on HIV prevention among young people in South Africa, was estimated to have reached 24% of the target population (with 43% reporting having engaged with MTV Shuga series more broadly) [25]. Though some other high profile examples, namely the fourth series of Soul City (SC4) in South Africa [33] and Sexto Sentido in Nicaragua [34], achieved even greater reach (approximately two-thirds of the national target population for SC4, and nine-tenths for Sexto Sentido), these interventions comprised nationally broadcast television *and* radio series which were part of long-running, already high profile and well-resourced national media campaigns. Data from the Sexto Sentido evaluation showed reach increasing steadily over the two-year follow-up period [34], showing the potential for a programme like TWG to increase its reach were the period of programming to be extended. Furthermore, the populations SC4 and Sexto Sentido served were more urban, more highly educated and had higher levels of television and radio ownership than those observed in our study villages. If we restrict our listenership estimates to those living in households with radios, TWG did in fact achieve comparable listenership levels (64% among men; 74% among women). It is also encouraging to note that our estimates of more intensive TWG exposure (listening most weeks/every week) are comparable to those for high exposure in the SC4 evaluation [33].

Our data revealed interesting demographic patterns in TWG listenership. Our finding that women were less likely than men to listen to TWG early on is similar to the pattern we observed in the original trial of the *SASA!* community mobilisation intervention in which we found men to be more heavily exposed than women [12]. Though contrary to some intervention studies that have found men harder to engage than women [25, 50], one hypothesis behind our original trial finding was that women may be disproportionately confined to their homes for a range of reasons including competing family demands, less formal employment outside the household, and lack of autonomy over their movement. While we surmised that such factors would present less of a barrier to engagement with a radio intervention, we did find that women sometimes mentioned household responsibilities (including those relating to childcare) as a barrier to listening. Lack of radio access (or control over radio use) may also have hindered women’s early engagement with TWG. It is encouraging to note, however, that by the endline survey, women had overcome this hurdle and were listening in equal numbers to men. It appears that women found ways to listen despite lack of radio ownership, for example listening with neighbours or partners. However, less educated women remained less likely than those with secondary or higher education to listen to TWG throughout the duration of the intervention. This was in part explained by lack of radio ownership, which they were perhaps less equipped to circumvent. We also hypothesise that TWG may have resonated less with less educated women, potentially exacerbated by the language issues highlighted in the qualitative research. These data provide a useful reminder that barriers to access to an intervention may differentially affect, or prove more insurmountable for some sub-groups of the target population, and that ways of mitigating such barriers should be considered during programme design (and subsequently monitored/addressed during implementation). This is particularly pertinent since these ‘excluded’ subgroups may also be those most vulnerable to IPV [51].

Our finding that listening levels among men increased steadily with age, and among those with a regular partner and children (characteristics which are highly correlated), is likely to reflect a greater perceived relevance of TWG to these men. Not only might storylines resonate more with this group, but we also observed men listening to TWG *alongside* their partners and children. Indeed, men without partners and/or children were those most likely to cite lack of relevance as a reason for stopping listening to TWG. Since evidence suggests that perpetration of IPV often begins at an early age, during adolescence, when expectations and beliefs about relationships are formed, it is crucial for interventions to find ways to more effectively target and engage younger men [52, 53].

A more commonly cited barrier to listening, particularly among men, was being ‘too busy’. However, the fact that TWG was aired at multiple time-slots on multiple stations was helpful in allowing people to overcome this barrier. Use of multiple TV channels and radio stations has also been used successfully by other mass media interventions to maximise coverage [34].

A high proportion of those who listened to TWG did so with others from their households and communities. Most commonly people reported having listened with their partners, but women were also particularly likely to have listened with neighbours and men to have listened with their children. This shared listening is important for a number of reasons. First, as already discussed, radio ownership was not universal in our study communities. Nevertheless, one of the ways in which those without radios managed to listen was through listening with others. Secondly, many people said listening with others encouraged them to start/continue listening to TWG. They would motivate or remind each other to listen, fill each other in on missed episodes, and help improve each other’s understanding of the programme. The fact that men often listened with their children also allowed TWG to reach children and adolescents in the community, providing the potential to engender more progressive norms and expectations around intimate relationships at a young age. Lastly, evidence suggests that communal exposure may be an important facilitator of norm change through a ‘conformity effect’, reinforcing participants’ sense that others in their communities or social networks are simultaneously changing their stance on the same beliefs [38, 54]. Participants in our study reported that spontaneous shared listening aided reflection on and discussion of issues raised, promoted accountability and consultation between partners, and created a sense of togetherness and support among women when content resonated with personal experiences. High levels of reported discussion with others about TWG also allows for diffusion (or extended reach) of messaging, regardless of whether or not it encourages others to listen to TWG. These potential mechanisms through which shared listening and discussion may facilitate change will be further explored in a subsequent publication.

Reported exposure to in-person *SASA! Together* programming was considerably lower than expected among survey respondents. It is worth noting that exposure to an intervention such as *SASA! Together* is difficult to measure due to the diverse and informal routes though which people can be exposed, often unknowingly, as they go about their daily lives (for example, CAs are trained to have informal ‘quick chats’ with their neighbours and other community members as part of the program implementation). Our measure of exposure incorporated attendance at *SASA! Together* in-person activities, having seen *SASA! Togethe*r materials (e.g., posters, logos), and/or having consulted with a Community Activist. While we may have underestimated exposure somewhat, our low exposure estimates more likely highlight the challenges around implementing this type of intervention across a large geographical area. Villages were geographically dispersed and not all easily accessible by road, with resource constraints impacting staffing, CA recruitment and retention, and monitoring of implementation (Hameed S, Musenze J, Awino E, Abramsky T, Witt A, Nakuti J, Buller A: Assessing Together with Gloria: A Process Evaluation of an edutainment-based intervention to prevent violence against women in Uganda, Submitted). Indeed, from the outset of the study, we were aware that some of our study villages no longer contained an active CA. These types of impediments to in-person programming, typical when community mobilisation approaches to VAW are ‘scaled up’ among geographically dispersed populations, were indeed a main impetus behind the development of the TWG radio drama. Our results demonstrate that TWG was successful in expanding the reach of *SASA! Together* community programming, both in terms of overall numbers reached and in terms of reducing geographical disparities in access.

There was evidence of an association between TWG and in-person *SASA! Together* exposure, with those who listened to TWG more regularly also more likely to have been exposed to in-person programming. This likely in part arises because those interested in the one also have a propensity to be interested in the other. However, there may also be a bidirectional influence, whereby each actively influences interest/participation in the other. Indeed, qualitative data indicates the presence of active synergies between the two, with CAs actively playing TWG episodes in public community settings.

This study has several limitations. Lack of a control group will limit the causal inference we are able to make from planned impact analyses. However, this has not impeded our current analysis of intervention reach. Our planned mid-line quantitative survey was cancelled due to an Ebola outbreak, meaning that our data on intervention ‘trajectory’ are less complete than originally proposed. Nevertheless, we still managed to collect data at two time-points, and diverted resources from the midline survey towards increasing our endline sample size. Though lack of a ‘pure’ baseline prior to intervention implementation will again have a bearing on what inferences can be made from future impact analyses, it has been beneficial in this analysis of reach to have estimates from the early weeks of implementation. Despite sampling from the same neighbourhoods in the two survey rounds, there were some significant demographic differences between our baseline and endline samples. This is something that we will have to adjust for in our analysis looking at change in outcomes over time, but does not detract from this analysis of intervention reach.

The study also has considerable strengths. As a proof-of-concept study, it was designed to assess the *potential* for a mass media intervention to boost the reach and impact of an in-person community-level VAW prevention intervention. We therefore collected detailed data on a wide range of process related indicators, using both quantitative and qualitative means to better understand *mechanisms* that may influence success. Interviewers underwent intensive training on the conduct of VAW research, thus enhancing both participant safety and data quality. Data from multiple time-points allowed us to assess how reach and factors influencing reach may change over time. Change in attitudinal and behavioural outcomes over time will also be explored in future mixed-methods analyses.

## Conclusions

TWG was widely listened to in our study communities, and retained the interest of listeners throughout the duration of programming. It expanded the overall reach of *SASA! Together* community programming, and reduced disparities in access. Synergistic effects between exposure to TWG and exposure to in-person programming were also observed. Barriers to listening included lack of radio access, which disproportionately affected women, and lack of time. People often listened with others in their community, providing a means of listening even if they didn’t have a radio in their own household, as well as promoting discussion and reflection on the issues raised. Future iterations of the intervention should consider additional ways of facilitating access, for example through communal broadcasts in public spaces. The use of multiple radio-stations and broadcasting time-slots was helpful in allowing people to listen despite competing demands on their time, and is a strategy that could be expanded upon in the future. Areas for potential development also include the incorporation of characters and storylines that resonate more with younger men without children, a key group to engage in the prevention of VAW. Having demonstrated the expansion in reach achieved by TWG, future analyses will include a process evaluation and an exploration of whether (and the mechanisms through which) listening to TWG is associated with changes in attitudes and behaviours related to IPV and intimate relationships more broadly.

## Supplementary Information

Additional file 1. *SASA! Together* – Community member survey questionnaire. *SASA! Together*/Together with Gloria evaluation – community member survey questionnaire.

Additional file 2. Timing and number of In-Depth Interviews (IDI) and Focus Group Discussions (FGD) conducted. Table showing the number of IDIs and FGDs conducted at Baseline, Midline and Endline

Additional file 3. Characteristics associated with exposure to in-person *SASA! Together* programming. Table of associations between village-level and socio-demographic characteristics and exposure to in-person *SASA! Together* programming.

## Data Availability

Data from the community surveys will be made available, upon request, via the London School of Hygiene and Tropical Medicine data repository (Data Compass) by the end of September 2024. The data will be available upon request to members of the scientific and medical community for non-commercial use only, on condition that they comply with the ethical obligations and consent conditions of the original study. Data requests submitted through the LSHTM Data Compass are automatically sent to the study team and LSHTM Research Data Management Service (a non-author contact). For additional queries, the LSHTM Research Data Management Service can be contacted by emailing researchdatamanagement@lshtm.ac.uk.The qualitative transcripts cover topics that are considered sensitive by participants and contain context-specific information that would enable them to be identified if transcripts were made available in their entirety. To safeguard the confidentiality and welfare of the individuals interviewed, we are therefore only able to share excerpts of anonymised transcripts that underpin the conclusions drawn in our manuscript. These will be made available via Data Compass under the same conditions as the quantitative data.

## Abbreviations

CA: Community activist
FGD: Focus group discussion
GPS: Global Positioning System
ICC: Intra-cluster correlation coefficient
IDI: In-depth interview
IPV: Intimate partner violence
IQR: Interquartile range
LSHTM: London School of Hygiene and Tropical Medicine
PVI: Peripheral Vision International
RWI: Research World International Ltd
SCT: Social Cognitive Theory
SC4: Soul City, fourth series (South African edutainment programme)
SLT: Social Learning Theory
TWG: Together with Gloria (*SASA! Together* radio drama)
UGANET: Uganda Network on Law Ethics and HIV/AIDS
VAW: Violence against women
