# Supplementary material for: To what extent does Together with Gloria! expand the reach of SASA! Together community programming? A mixed methods evaluation of an edutainment intervention in Kasese, Uganda
Source: BMC Public Health. 2025 Sep 29;25:3141. doi: 10.1186/s12889-025-24178-x (PMC12481820; doi:10.1186/s12889-025-24178-x)
Supplement: Supplementary file 1 — Additional file 1. SASA! Together – Community member survey questionnaire. SASA! Together/Together with Gloria evaluation – community member survey questionnaire. [file 12889_2025_24178_MOESM1_ESM.docx]

| **No.** | **Questions** | **Response categories** | **Response codes** |
| --- | --- | --- | --- |
|  | | | |
| **Sociodemographics**  In this first section, I would like to ask you a little bit about yourself and your living situation. | | | |
| Q1 | INTERVIEWER: RECORD THE RESPONDENT’S SEX | Female | 2 |
|  |  | Male | 1 |
| Q2 | INTERVIEWER: RECORD IF THE RESPONDENT HAS ALBINISM [DO NOT ASK RESPONDENT DIRECTLY] | Albinism | 1 |
|  |  | No albinism | 0 |
| Q3 | What year were you born in?  [IF EXACT YEAR NOT KNOWN, ASK RESPONDENT TO GIVE ESTIMATED YEAR]  [RECORD 9999 FOR DON’T KNOW/NO ESTIMATE] | Actual year [ ][ ][ ][ ] | Date format  9999=Don’t know/no estimate |
|  |  | Estimated year [ ][ ][ ][ ] |  |
| Q4 | *If ‘Don’t know’ year of birth:*  How old are you?  [IF EXACT AGE NOT KNOWN, ASK RESPONDENT TO GIVE ESTIMATED AGE]  [RECORD 9999 FOR DON’T KNOW/NO ESTIMATE] | Actual age [x][x] | Numeric  9999=Don’t know/no estimate |
|  |  | Estimated age [x][x] |  |
| Q5 | How long have you lived in this community? | Less than 6 months | 1 |
|  |  | >6 months but <1 year | 2 |
|  |  | 1-3 years | 3 |
|  |  | 4-5 years | 4 |
|  |  | >5 years | 5 |
|  |  | Don’t know/no estimate | 99 |
| Q6 | During the last *3 months*, have you actually stayed most of the time in another town/village, such as for work, school or staying with other family? | Yes | 1 |
|  |  | No | 0 |
| Q7 | What language do you speak the most at home? | Rukhonzo | 1 |
|  |  | Runyakitara | 2 |
|  |  | Rusongora | 3 |
|  |  | Rutooro | 4 |
|  |  | Other (specify) | 5 |
|  | *If othe*r: please specify | [x] | Free text |
| *Q8* | *What is your faith/religion?* | Muslim | 1 |
|  |  | Bahai | 2 |
|  |  | Catholic | 3 |
|  |  | Pentecostal | 4 |
|  |  | Seventh day Adventist | 5 |
|  |  | Anglican | 6 |
|  |  | Born Again | 7 |
|  |  | Hindu | 8 |
|  |  | Other religion (specify) | 9 |
|  |  | No religion | 10 |
|  | *If other:* please specify | [x] | Free text |
| Q9 | What is the highest level of school you completed? | None | 1 |
|  |  | Some primary | 2 |
|  |  | All primary | 3 |
|  |  | Some secondary (but below O level) | 4 |
|  |  | Form 4 (O level) | 5 |
|  |  | Form 6 (A level) | 6 |
|  |  | Vocational training | 7 |
|  |  | Other tertiary institution | 8 |
|  |  | University | 9 |
| Q10 | Can you read? | Yes, without difficulty | 1 |
|  |  | Yes, with difficulty | 2 |
|  |  | No | 3 |
|  |  | Prefer not to say | 4 |
| Q11 | What are the main sources of income for you and your household?  SELECT ALL THAT APPLY | Money from own work/business | Tick boxes  ‘Other, specify’ is free text |
|  |  | Money from husband/partner’s work/business |  |
|  |  | Support from other relatives |  |
|  |  | Pension |  |
|  |  | Social services/welfare |  |
|  |  | Other (specify) |  |
|  | *If other:* specify | [x] | Free text |
| Q12 | In the past 3 months, have you personally done any work for cash or in-kind payment?  [IF YES, PROBE WHETHER FOR CASH OR IN-KIND PAYMENT] | No | 0 |
|  |  | Yes – cash | 1 |
|  |  | Yes – in-kind | 2 |
|  |  | Yes – cash and in-kind | 3 |
| Q13 | *If yes:*  What is the main type of work you have been doing in the past 3 months?  [IF RESPONDENT HAS MULTIPLE MAIN SOURCES OF INCOME, SELECT ALL THAT APPLY] | Unskilled manual work (construction, porter) | Tick boxes  ‘Other, specify’ is free text |
|  |  | Farming |  |
|  |  | Fishing or fishmonger |  |
|  |  | Petty business/trader (market vendor, fishmonger, chapattis /tea /porridge /snack seller) |  |
|  |  | Transport (boda-boda, cyclist, taxi driver, bus/taxi conductor, big truck driver, private driver/special hire) |  |
|  |  | Service industry (domestic worker, restaurant/bar worker) |  |
|  |  | Skilled manual work (carpenter, mechanic, electrician, plumber) |  |
|  |  | Formal business/trader (shop keeper, barber, events management) |  |
|  |  | Professional work (teacher, banker, doctor/health worker, social worker) |  |
|  |  | Landlord/rent money |  |
|  |  | Other: specify |  |
|  | *If other:* please specify | [x] |  |
| Q14 | INTERVIEWER: RECORD WHAT THE ROOF OF THE HOUSE IS MADE OF  [OBSERVE AND RECORD] | Tiled or concrete roof | 1 |
|  |  | Corrugated iron | 2 |
|  |  | Roof from natural materials | 3 |
|  |  | Rudimentary roof (plastic/carton) | 4 |
|  |  | Other (specify) | 5 |
|  | *If other*: please specify | [x] | Free text |
| Q15 | INTERVIEWER: RECORD WHETHER THE HOUSEHOLD IS IN GATED COMPOUND  [OBSERVE AND RECORD] | Gated | 1 |
|  |  | Not gated | 0 |
| If you don’t mind, I would like to ask you a few questions about your household. | | | |
| Q16 | What is the main source of drinking water for your household? | Inside tap/piped water in residence | 1 |
|  |  | Outside tap (piped water) within household compound | 2 |
|  |  | Public tap | 3 |
|  |  | Outside/public well/borehole | 4 |
|  |  | Spring water | 5 |
|  |  | River/stream/pond/lake/dam (surface water) | 6 |
|  |  | Rainwater | 7 |
|  |  | Tanker/truck water vendor | 8 |
|  |  | Bottled water | 9 |
|  |  | Other (specify) | 10 |
|  |  | Refused/Prefer not to answer | 11 |
|  | *If other:* please specify | [x] | Free text |
| Q17 | What kind of toilet facility does your household have?  [IF MORE THAN ONE, SELECT HIGHEST ON LIST] | Shared flush toilet | 1 |
|  |  | Own flush toilet | 2 |
|  |  | Shared pit latrine | 3 |
|  |  | Ventilated improved pit latrine | 4 |
|  |  | Traditional pit latrine/latrine | 5 |
|  |  | Composting toilet | 6 |
|  |  | River/canal | 7 |
|  |  | No facility/bush/field | 8 |
|  |  | Other (specify) | 9 |
|  |  | Refused/Prefer not to answer | 10 |
|  | *If other:* please specify | [x] | Free text |
| Q18a | Does your household have: | | |
|  | Electricity | Yes | 1 |
|  |  | No | 0 |
|  |  | Do not know | 99 |
|  | A radio in working condition | Yes | 1 |
|  |  | No | 0 |
|  |  | Do not know | 99 |
|  | A television in working condition | Yes | 1 |
|  |  | No | 0 |
|  |  | Do not know | 99 |
|  | A computer | Yes | 1 |
|  |  | No | 0 |
|  |  | Do not know | 99 |
|  | A non-mobile telephone/land line | Yes | 1 |
|  |  | No | 0 |
|  |  | Do not know | 99 |
| Q18b | Does any adult member of your household own: | | |
|  | A bicycle | Yes | 1 |
|  |  | No | 0 |
|  |  | Do not know | 99 |
|  | A motorcycle or motor scooter | Yes | 1 |
|  |  | No | 0 |
|  |  | Do not know | 99 |
|  | A car or truck | Yes | 1 |
|  |  | No | 0 |
|  |  | Do not know | 99 |
|  | An animal-drawn truck | Yes | 1 |
|  |  | No | 0 |
|  |  | Do not know | 99 |
|  | A functioning mobile phone | Yes | 1 |
|  |  | No | 0 |
|  |  | Do not know | 99 |
|  | *If ‘Yes’:*  Is this a smart phone or a regular mobile phone? | Smart phone | 1 |
|  |  | Regular phone | 2 |
|  |  | Both types in household | 3 |
|  |  | Don’t know | 99 |
| Q19 | Does someone in your household own this house or is it rented? | Own | 1 |
|  |  | Rent | 2 |
|  |  | Caretaker | 3 |
|  |  | Provided by job | 4 |
|  |  | Refused | 98 |
|  |  | Do not know | 99 |
| **Personal relationships**  I would now like to learn a little more about you and your family. | | | |
| Q21 | What is your current relationship status?  [PLEASE READ EACH OPTION AND CHOOSE ONE] | Married | 1 |
|  |  | Have a regular partner and living together | 2 |
|  |  | Have a regular partner but not living together | 3 |
|  |  | Have a casual sexual partner | 4 |
|  |  | Temporarily separated | 5 |
|  |  | Divorced | 6 |
|  |  | Widowed | 7 |
|  |  | Single | 8 |
|  | *Q22. If currently in a relationship (ie married, living as married, have a regular partner):*  How long have you been in this relationship?  [ANSWER IN YEARS AND MONTHS] | Years [ ][ ]  Months [ ][ ] | Numeric  Months (1-12)  Don’t know=999 |
|  | *Q23. For female respondents, if currently married/living together with partner:*  Does your husband/partner have other wives or does he live with other women as if married? | Yes | 1 |
|  |  | No | 0 |
|  |  | Do not know | 99 |
|  |  | Refused to answer | 98 |
|  | *Q24. For male respondents, if currently married/living together with partner:*  Altogether, how many wives do you have, or partners that you live with, as if married?  [CATEGORISE RESPONSE AS ‘ONE’ OR ‘MORE THAN ONE’] | One wife/live in partner | 1 |
|  |  | More than one wife/live in partner | 2 |
|  |  | Do not know | 99 |
|  |  | Refused to answer | 98 |
|  | *Q25. If female and NOT currently in a relationship:*  In the last 3 months have you been married, lived together with a man as if married, or had a regular partner? | Yes | 1 |
|  |  | No | 0 |
|  |  | Prefer not to say | 97 |
|  | *Q26. If male and NOT currently in a relationship:*  In the last 3 months have you been married, lived together with a woman as if married, or had a regular partner? | Yes | 1 |
|  |  | No | 0 |
|  |  | Prefer not to say | 97 |
|  | *Q25B/Q26B. If NOT currently in a relationship but in a relationship in last 3 months:*  How long ago did this relationship end?  [ANSWER IN MONTHS] | [x][x] | Numeric (1-3) |
|  | *Q25C. If female, NOT currently in a relationship and NOT in a relationship in last 3 months:*  Have you ever been married, lived together with a man as if married, or had a regular partner? | Yes | 1 |
|  |  | No | 0 |
|  |  | Prefer not to say | 97 |
|  | *Q26C. If male, NOT currently in a relationship and NOT in a relationship in last 3 months:*  Have you ever been married, lived together with a woman as if married, or had a regular partner? | Yes | 1 |
|  |  | No | 0 |
|  |  | Prefer not to say | 97 |
|  | Q27. How many children up to the age of 18 are you responsible for? Please tell us how many children live in your household and how many are living elsewhere. | [x total]  [x number living with you]  [x number living elsewhere] | Numeric |
|  | *Q28. If responsible for at least one child:*  How many of these children up to the age of 18 are enrolled in school? | [x][x] | Numeric |
| **Disability**  Everyone’s body and mind work differently. In the next few questions, we’d like to learn about how your body/mind works. We would like to start by asking about difficulties you may have doing certain activities because of a health problem. Please remember that this information is confidential and you may choose to skip any question you do not want to answer. | | | |
| Q29 | Do you have difficulty seeing, even if wearing glasses?  [READ RESPONSE OPTIONS ALOUD] | No, no difficulty | 0 |
|  |  | Yes, some difficulty | 1 |
|  |  | Yes, a lot of difficulty | 2 |
|  |  | Cannot do it at all | 3 |
|  |  | [Refused to answer] | 98 |
| Q30 | Do you have difficulty hearing, even if using a hearing aid?  [READ RESPONSE OPTIONS ALOUD] | No, no difficulty | 0 |
|  |  | Yes, some difficulty | 1 |
|  |  | Yes, a lot of difficulty | 2 |
|  |  | Cannot do it at all | 3 |
|  |  | [Refused to answer] | 98 |
| Q31 | Do you have difficulty walking or climbing steps?  [READ RESPONSE OPTIONS ALOUD] | No, no difficulty | 0 |
|  |  | Yes, some difficulty | 1 |
|  |  | Yes, a lot of difficulty | 2 |
|  |  | Cannot do it at all | 3 |
|  |  | [Refused to answer] | 98 |
| Q32 | Do you have difficulty remembering or concentrating?  [READ RESPONSE OPTIONS ALOUD] | No, no difficulty | 0 |
|  |  | Yes, some difficulty | 1 |
|  |  | Yes, a lot of difficulty | 2 |
|  |  | Cannot do it at all | 3 |
|  |  | [Refused to answer] | 98 |
| Q33 | Do you have difficulty (with caring for yourself such as) bathing or dressing yourself?  [READ RESPONSE OPTIONS ALOUD] | No, no difficulty | 0 |
|  |  | Yes, some difficulty | 1 |
|  |  | Yes, a lot of difficulty | 2 |
|  |  | Cannot do it at all | 3 |
|  |  | [Refused to answer] | 98 |
| Q34 | Using your usual language, do you have difficulty communicating (for example understanding or being understood by others)?  [READ RESPONSE OPTIONS ALOUD] | No, no difficulty | 0 |
|  |  | Yes, some difficulty | 1 |
|  |  | Yes, a lot of difficulty | 2 |
|  |  | Cannot do it at all | 3 |
|  |  | [Refused to answer] | 98 |
| Q35 | How often do you feel worried, nervous or anxious? | Daily | 4 |
|  |  | Weekly | 3 |
|  |  | Monthly | 2 |
|  |  | A few times a year | 1 |
|  |  | Never | 0 |
|  |  | [Refused to answer] | 98 |
| Q36 | *If not ‘Never’ to how often worried/anxious:*  Thinking about the last time you felt worried, nervous or anxious, how would you describe the level of these feelings? | A little | 1 |
|  |  | A lot | 2 |
|  |  | Somewhere in between a little and a lot | 3 |
|  |  | [Refused to answer] | 98 |
| Q37 | How often do you feel depressed? | Daily | 4 |
|  |  | Weekly | 3 |
|  |  | Monthly | 2 |
|  |  | A few times a year | 1 |
|  |  | Never | 0 |
|  |  | [Refused to answer] | 98 |
| Q38 | *If not ‘Never to how often depressed:*  Thinking about the last time you felt depressed, how depressed did you feel? | A little | 1 |
|  |  | A lot | 2 |
|  |  | Somewhere in between a little and a lot | 3 |
|  |  | [Refused to answer] | 98 |
| **Exposure to SASA! Together**  I would now like to ask you a few questions about a programme that you may have heard about, seen or participated in, in your community. | | | |
| Q39 | Have you ever heard of a programme called *SASA! Together*? | Yes | 1 |
|  |  | No | 0 |
| Q40 | I will now show you some examples of some *SASA! Together* materials. Have you ever seen any *SASA! Together* **materials** about violence against women and relationships between men and women? [*If yes,* *probe:* how many times have you seen materials like this?]  [SHOW/DESCRIBE TO RESPONDENT EXAMPLE MATERIALS – E.G. LEAFLETS, POSTERS, INFORMATION SHEETS, GAMES, SOCIAL MEDIA TILES] | No, Never | 0 |
|  |  | Yes - Once | 1 |
|  |  | Yes - A few 2-5 times | 2 |
|  |  | Yes - Many 5+ times | 3 |
| Q41 | Have you ever been to a **community activity** organised by a *SASA! Together* Community Activist, where you talked about violence against women and relationships between women and men? [*If yes, probe:* how many times have you been to these types of activities?]    [PROVIDE RESPONDENT WITH EXAMPLES OF ACTIVITIES SUCH AS:  - COMMUNITY CONVERSATION  - POWER POSTER DISCUSSION  - DRAMA SKETCH  - DEEPER DISCUSSION  - QUICK CHAT WITH A LEADER/ACTIVIST IN YOUR COMMUNITY] | No, Never | 0 |
|  |  | Yes - Once | 1 |
|  |  | Yes - A few 2-5 times | 2 |
|  |  | Yes - Many 5+ times | 3 |
|  | Now I would like to ask you about other ways you may have been involved in activities related to SASA! Together. How many times have you done any of the following things? | |  |
| Q42 | Sought advice from a SASA! Together community activist, leader or ally | Never | 0 |
|  |  | Once | 1 |
|  |  | A few 2-5 times | 2 |
|  |  | Many 5+ times | 3 |
|  |  | N/A | 96 |
| **Radio exposure**  I now have a few questions about how you access media and information. | | | |
| Q43 | Thinking about the past 3 months, how often would you say you have listened to the radio? | Most days | 4 |
|  |  | At least once per week | 3 |
|  |  | At least once per month | 2 |
|  |  | Less than once per month | 1 |
|  |  | Never | 0 |
|  |  | Do not know | 99 |
| Q44 | *If not ‘Never’ to radio:*  Do you usually listen to the radio on a traditional radio or a mobile phone?  [IF ‘RADIO’, PROBE TO FIND OUT POWER SOURCE] | Radio (battery operated) | 1 |
|  |  | Radio (mains power supply) | 2 |
|  |  | Radio (solar charging) | 3 |
|  |  | Mobile phone | 4 |
|  |  | Both radio and mobile phone | 5 |
| Q45 | *If not ‘Never’ to radio:*  Who does the radio/phone you usually listen to belong to? | My household (shared) | 1 |
|  |  | Me personally | 2 |
|  |  | My partner (living in my household) | 3 |
|  |  | Other member of my household | 4 |
|  |  | My partner (not living in my household) | 5 |
|  |  | Extended family (not living in my household) | 6 |
|  |  | Friend or neighbour | 7 |
|  |  | Workplace | 8 |
|  |  | School or college | 9 |
|  |  | Church, Mosque or other place of worship | 10 |
|  |  | Community leader/office of community leader | 11 |
|  |  | *SASA! Together* Community Activist | 12 |
|  |  | Other public venue | 13 |
|  |  | Other (specify) | 14 |
|  | *If other:* please specify | [x] | Free text |
| Q46 | *If not ‘Never’ to radio:*  Which radio stations do you listen to?  [PLEASE SELECT ALL THAT APPLY] | UBC Ngeya | Tick boxes |
|  |  | Kasese Guide Radio |  |
|  |  | Messiah Radio |  |
|  |  | Light FM Radio |  |
|  |  | Other (specify) |  |
|  | *If other:* please specify | [x] | Free text |
| Q47 | In the past 3 months, other than radio, which of the following have you used to access media and information?  [SELECT ALL THAT APPLY] | Television | Tick boxes |
|  |  | Newspapers/magazines |  |
|  |  | Social media (e.g. WhatsApp, Facebook, Instagram, etc) |  |
|  |  | Online news sites/other websites |  |
|  |  | Other (specify) |  |
|  | *If other:* please specify | [x] | Free text |
| **Exposure to *SASA! Together* Radio Booster**  I now have some questions about a radio programme you may have heard about or listened to in recent months. Please remember that there are no right or wrong answers. | | | |
| Q48 | Have you heard of the *SASA! Together* radio program, ‘Together with Gloria’? | Yes | 1 |
|  |  | No | 0 |
| Q49 | *If have heard of Together with Gloria:*  How did you first hear about ‘Together with Gloria’?  [SELECT ALL THAT APPLY] | Advert on the radio | Tick boxes |
|  |  | Social media (eg Whatsapp, Facebook, Instagram) |  |
|  |  | Newspaper |  |
|  |  | SASA! Together community activist/leader/ally |  |
|  |  | SASA! Together activity |  |
|  |  | Friend |  |
|  |  | Family member |  |
|  |  | Neighbour |  |
|  |  | Community leader(s) |  |
|  |  | Church/Mosque/other place of worship |  |
|  |  | Other (specify) |  |
|  |  | Don’t remember |  |
|  | *If other:* please specify | [x] | Free text |
| Q50 | *If have heard of Together with Gloria:*  Have you ever listened to ‘Together with Gloria’? | Yes | 1 |
|  |  | No | 0 |
|  |  | Do not know | 99 |
| Q51 | *If have heard of Together with Gloria but have not listened to it:*  We are interested if there are any specific reasons why you have never listened to ‘Together with Gloria’.  Please tell me if any of the following reasons apply to you.  [SELECT ALL THAT APPLY] | I was too busy | Tick boxes |
|  |  | It was played at an inconvenient time for me |  |
|  |  | It was played at a time that clashed with another radio programme |  |
|  |  | The content sounded boring |  |
|  |  | The content did not sound relevant to me or my life |  |
|  |  | The content sounded offensive to me |  |
|  |  | No one else I know listened to it |  |
|  |  | I no longer had radio access |  |
|  |  | I didn’t think my partner or family members would approve of me listening to it |  |
|  |  | I was afraid of being overheard listening to it/my partner stopped me from listening |  |
|  |  | No specific reason  *[Can’t be selected in combination with any other answer]* |  |
|  |  | Other (specify) |  |
|  | *If other:* please specify | [x] | Free text |
| Q52 | *If have ever listened:*  Together with Gloria has now finished airing, and we are interested in how many people were listening to the final few episodes.  Have you listened to any Together with Gloria episodes since Christmas? | Yes | 1 |
|  |  | No | 0 |
| Q53 | *If have ever listened but not listened since Christmas:*  Why did you stop listening to Together with Gloria?  [SELECT ALL THAT APPLY] | I was too busy | Tick boxes |
|  |  | It was played at an inconvenient time for me |  |
|  |  | It was played at a time that clashed with another radio programme |  |
|  |  | The content was boring |  |
|  |  | The content was not relevant to me or my life |  |
|  |  | The content was offensive to me |  |
|  |  | The episodes were played too frequently |  |
|  |  | The episodes were not played frequently enough |  |
|  |  | The episodes were too long |  |
|  |  | The episodes were too short |  |
|  |  | No one else I know listened to it |  |
|  |  | I no longer had radio access |  |
|  |  | I didn’t think my partner or family members would approve of me listening to it |  |
|  |  | I was afraid of being overheard listening to it/my partner stopped me from listening |  |
|  |  | There was a family crisis |  |
|  |  | I was sick |  |
|  |  | Other (specify) |  |
|  | *If other*: please specify | [x] | Free text |
|  |  |  |  |
| Q55 | *If have ever listened to Together with Gloria :*  Which, if any, of the following characters interested you the most?  [SELECT ALL THAT APPLY] | Gloria | Tick boxes |
|  |  | Tata Gloria |  |
|  |  | Mama Gloria |  |
|  |  | Joy (Gloria’s best friend) |  |
|  |  | Ali (Gloria’s boss and friend) |  |
|  |  | Jack (Gloria’s boyfriend) |  |
|  |  | Adam (bad guy) |  |
|  |  | Managing Director (radio station) |  |
|  |  | Mr Mshindi |  |
|  |  | Mrs Mshindi |  |
|  |  | Margaret (*SASA! Together* activist) |  |
|  |  | Mama Musa |  |
|  |  | Radio show callers (women) |  |
|  |  | Radio show callers (men) |  |
|  |  | Other (specify) |  |
|  |  | None *[Can’t be selected in combination with any other answer]* |  |
|  |  | Do not know |  |
|  | *If other:* please specify |  |  |
| Q56 | *If have ever listened to Together with Gloria:*  Since ‘Together with Gloria’ started there have been 33 episodes. How many do you think you have listened to? | Very few episodes | 1 |
|  |  | Some episodes | 2 |
|  |  | Most episodes | 3 |
|  |  | All episodes | 4 |
|  |  | Do not know | 99 |
| Q57 | *If have ever listened to Together with Gloria:*  Since you started listening to ‘Together with Gloria’, would you say you listened… | Every week | 4 |
|  |  | Most weeks | 3 |
|  |  | From time to time | 2 |
|  |  | Hardly ever | 1 |
|  |  | Do not know | 99 |
| Q58 | *If have ever listened to Together with Gloria:*  Which radio station did you usually listen to ‘Together with Gloria’ on?  [SELECT ALL THAT APPLY] | UBC Ngeya | Tick boxes |
|  |  | Kasese Guide Radio |  |
|  |  | Messiah Radio |  |
|  |  | At listener group/with Community Activist |  |
|  |  | Other (specify) |  |
|  |  | Do not know *[Can’t be selected in combination with any other answer]* |  |
|  | *If other:* please specify | [x] | Free text |
| Q59 | *If select UBC Ngeya, Kasese Guide Radio, Messiah Radio or Do not know:*  Which broadcasting/time-slot did you usually listen to ‘Together with Gloria’ in?  [SELECT THE SLOTS RESPONDENT USUALLY LISTENS IN. YOU MAY SELECT MORE THAN ONE] | *If UBC Ngeya:* | Tick boxes |
|  |  | Monday 3.30pm |  |
|  |  | Wednesday 3.30pm |  |
|  |  | Friday 8.15pm |  |
|  |  | Do not know/remember |  |
|  |  | *If Kasese Guide Radio:* |  |
|  |  | Monday 8.30pm |  |
|  |  | Tuesday 6.30pm |  |
|  |  | Friday 6.30pm |  |
|  |  | Do not know/remember |  |
|  |  | *If Messiah Radio:* |  |
|  |  | Monday 8.30pm |  |
|  |  | Wednesday 10am |  |
|  |  | Friday 8.30pm |  |
|  |  | Do not know/remember |  |
|  |  | *If Don’t know:* |  |
|  |  | Monday 3.30pm |  |
|  |  | Monday 8.30pm |  |
|  |  | Tuesday 6.30pm |  |
|  |  | Wednesday 10am |  |
|  |  | Wednesday 3.30pm |  |
|  |  | Friday 6.30pm |  |
|  |  | Friday 8.15pm |  |
|  |  | Friday 8.30pm |  |
|  |  | Do not know/remember |  |
| Q60 | *If have ever listened to Together with Gloria:*  Who did you usually listen to ‘Together with Gloria’ with?  [IF IT VARIES, ASK RESPONDENT TO CHOOSE THE MOST COMMON WAY THEY LISTEN] | By myself | 1 |
|  |  | With others from my household | 2 |
|  |  | With others from outside my household | 3 |
|  |  | With people from my household and other household(s) | 4 |
| Q61 | *If did not listen alone:*  Which of the following people have you listened to ‘Together with Gloria’ with?  [PLEASE SELECT ALL THAT APPLY] | Your partner | Tick boxes |
|  |  | A parent |  |
|  |  | An in-law |  |
|  |  | A sibling |  |
|  |  | Other extended family |  |
|  |  | A friend or neighbour |  |
|  |  | A child |  |
|  |  | Someone you work with |  |
|  |  | Someone in your faith community (Church, Mosque or other place of worship) |  |
|  |  | A community leader (including religious leaders) |  |
|  |  | SASA! Together listener group members/participants at a SASA! Together community activity |  |
|  |  | Other (specify) |  |
|  | *If other:* please specify | [x] | Free text |
| Q62 | *If do not listen alone:*  Thinking about the other people you have listened to ‘Together with Gloria’ with, have any of them been:  [SELECT ALL THAT APPLY] | Girls under 18 | Tick boxes |
|  |  | Boys under 18 |  |
|  |  | Women aged 18-29 |  |
|  |  | Women aged 30-50 |  |
|  |  | Women aged 50+ |  |
|  |  | Men aged 18-29 |  |
|  |  | Men aged 30-50 |  |
|  |  | Men 50+ |  |
| Q63 | *If have ever listened to Together with Gloria:*  Have you ever discussed the radio programme ‘Together with Gloria’ with other people in your community? | Yes | 1 |
|  |  | No | 0 |
| Q64 | *If have discussed Radio Programme:*  Who have you discussed ‘Together with Gloria’ with? Please select all that apply from the following list: | Your partner | Tick boxes |
|  |  | A parent |  |
|  |  | An in-law |  |
|  |  | A sibling |  |
|  |  | Other extended family |  |
|  |  | A friend or neighbour |  |
|  |  | A child |  |
|  |  | Someone you work with |  |
|  |  | Someone in your faith community (Church, Mosque or other place of worship) |  |
|  |  | A community leader (including religious leaders) |  |
|  |  | SASA! Together community activist, leader or ally |  |
|  |  | SASA! Together listener group members/participants at a SASA! Together community activity |  |
|  |  | Other (specify) |  |
|  | *If other:* please specify | [x] | Free text |
| Q65 | *If have ever listened to Together with Gloria:*  Did you ever recommend the radio programme ‘Together with Gloria’ to other people in your community? | Yes | 1 |
|  |  | No | 0 |
| Q66 | *If have recommended Together with Gloria:*  Who did you recommend the radio program to?  [SELECT ALL THAT APPLY] | Your partner | Tick boxes |
|  |  | A parent |  |
|  |  | An in-law |  |
|  |  | A sibling |  |
|  |  | Other extended family |  |
|  |  | A friend or neighbour |  |
|  |  | A child |  |
|  |  | Someone you work with |  |
|  |  | Someone in your faith community (Church, Mosque or other place of worship) |  |
|  |  | A community leader (including religious leaders) |  |
|  |  | Other people at a SASA! Together community activity |  |
|  |  | Other (specify) |  |
|  | *If other:* please specify | [x] | Free text |
| Q67 | *If have ever listened to Together with Gloria:*  We are interested in whether you found yourself thinking about the radio programme ‘Together with Gloria’ in between episodes. Would you say you thought about Gloria and the other characters in the radio drama: | Often | 1 |
|  |  | Sometimes | 2 |
|  |  | Rarely/Never | 3 |
| **Attitudes towards relationships**  In this community and elsewhere, people have different ideas about families and what is acceptable behavior for men and women in the home. We would like to know what you think about these things. | | | |
|  | When I read the following statements can you please say whether you personally agree or disagree: | |  |
| Q68 | A man’s pleasure is more important than a woman’s during sex | I strongly agree | 1 |
|  |  | I agree | 2 |
|  |  | I disagree | 3 |
|  |  | I strongly disagree | 4 |
| Q69 | It is acceptable for a married woman to ask her husband to use a condom | I strongly agree | 1 |
|  |  | I agree | 2 |
|  |  | I disagree | 3 |
|  |  | I strongly disagree | 4 |
| Q70 | It is acceptable for a married man to force his wife to have sex with him | I strongly agree | 1 |
|  |  | I agree | 2 |
|  |  | I disagree | 3 |
|  |  | I strongly disagree | 4 |
|  | In your opinion, is it okay for a married woman to refuse to have sex with her husband if: | |  |
| Q71.1 | She doesn’t want to | Yes | 1 |
|  |  | No | 0 |
| Q71.2 | He is drunk | Yes | 1 |
|  |  | No | 0 |
| Q71.3 | She is sick | Yes | 1 |
|  |  | No | 0 |
| Q71.4 | He mistreats her | Yes | 1 |
|  |  | No | 0 |
| Q71.5 | She suspects he is unfaithful | Yes | 1 |
|  |  | No | 0 |
| Q71.6 | She knows he is unfaithful | Yes | 1 |
|  |  | No | 0 |
| Q71.7 | She knows/suspects he is HIV positive | Yes | 1 |
|  |  | No | 0 |
| Q71.8 | He refuses to use a condom | Yes | 1 |
|  |  | No | 0 |
|  | When I read the following statements can you please say whether you personally agree or disagree: | |  |
| Q72.1 | A wife should obey her husband’s wishes even if she disagrees | I strongly agree | 1 |
|  |  | I agree | 2 |
|  |  | I disagree | 3 |
|  |  | I strongly disagree | 4 |
| Q72.2 | It is natural and right for a husband to decide whether or not his wife can work outside the home | I strongly agree | 1 |
|  |  | I agree | 2 |
|  |  | I disagree | 3 |
|  |  | I strongly disagree | 4 |
| Q72.3 | It is natural and right that a husband should decide who his wife can spend time with | I strongly agree | 1 |
|  |  | I agree | 2 |
|  |  | I disagree | 3 |
|  |  | I strongly disagree | 4 |
| Q72.4 | It is natural and right that a husband can decide what his wife wears in public | I strongly agree | 1 |
|  |  | I agree | 2 |
|  |  | I disagree | 3 |
|  |  | I strongly disagree | 4 |
| Q72.5 | A wife should have equal say in decisions about how household money is spent. | I strongly agree | 1 |
|  |  | I agree | 2 |
|  |  | I disagree | 3 |
|  |  | I strongly disagree | 4 |
| Q72.6 | It is a husband’s responsibility to be the primary provider for his family | I strongly agree | 1 |
|  |  | I agree | 2 |
|  |  | I disagree | 3 |
|  |  | I strongly disagree | 4 |
| Q72.7 | Both a husband and a wife have an equal responsibility for taking care of their children at home (e.g. bathing, feeding, dressing, caring for when sick, etc) | I strongly agree | 1 |
|  |  | I agree | 2 |
|  |  | I disagree | 3 |
|  |  | I strongly disagree | 4 |
| **Attitudes towards acceptability of Intimate Partner Violence (IPV)**  Husbands and wives sometimes have disagreements that end up in physical fights. People have different ideas about what is acceptable behaviour for men and women in these situations. | | | |
|  | In your opinion, does a man have a good reason to hit his wife if: | |  |
| Q73.1 | She disobeys him | Yes | 1 |
|  |  | No | 0 |
| Q73.4 | He suspects that she is unfaithful | Yes | 1 |
|  |  | No | 0 |
| Q73.5 | He finds out that she has been unfaithful | Yes | 1 |
|  |  | No | 0 |
| Q73.6 | She talks with neighbours about personal or family matters | Yes | 1 |
|  |  | No | 0 |
| Q73.7 | She neglects taking care of the children | Yes | 1 |
|  |  | No | 0 |
| Q73.8 | She does not complete household work to his satisfaction | Yes | 1 |
|  |  | No | 0 |
| Q73.9 | She refuses to have sexual relations with him | Yes | 1 |
|  |  | No | 0 |
| Q73.10 | She accuses him of infidelity | Yes | 1 |
|  |  | No | 0 |
|  | When I read the following statements can you please say whether you personally agree or disagree: | |  |
| Q74.1 | Women are sometimes to blame for violence against them | I strongly agree | 1 |
|  |  | I agree | 2 |
|  |  | I disagree | 3 |
|  |  | I strongly disagree | 4 |
| Q74.2 | Violence between husband and wife is a private matter and others should not intervene | I strongly agree | 1 |
|  |  | I agree | 2 |
|  |  | I disagree | 3 |
|  |  | I strongly disagree | 4 |
| Q74.3 | A woman should tolerate violence in order to keep her family together | I strongly agree | 1 |
|  |  | I agree | 2 |
|  |  | I disagree | 3 |
|  |  | I strongly disagree | 4 |
| Q74.4 | Sometimes a man uses violence to show his love | I strongly agree | 1 |
|  |  | I agree | 2 |
|  |  | I disagree | 3 |
|  |  | I strongly disagree | 4 |
| Q74.5 | Men can’t help being violent sometimes because it is in their nature | I strongly agree | 1 |
|  |  | I agree | 2 |
|  |  | I disagree | 3 |
|  |  | I strongly disagree | 4 |
| **Your relationship**  *[This whole section (Your relationship, Trust, Communication, Decision Making) only applies to respondents who have been in a relationship in last 3 months:*  *i.e. If response to ‘What is your current relationship status?’ is ‘Married’, ‘Regular partner and living together’, ‘Regular partner but not living together’*  *OR*  *If responded ‘Yes’ to ‘In the last 3 months have you been married, lived together with a man/woman as if married, or had a regular partner?’]*  The next few questions are about your relationship with your current/most recent partner. If anyone interrupts us I will change the topic of conversation. I would again like to assure you that your answers will be kept secret, and that you do not have to answer any question that you do not want to. May I continue?  We would like you to think back to how your relationship has been over the last 3 months.  [INTERVIEWER: FOR MALE RESPONDENTS IN A POLYGAMOUS RELATIONSHIP, ASK IN RELATION TO NEWEST RELATIONSHIP] | | | |
| Q75 | In general, how satisfied have you been with your relationship over the last 3 months? | Very satisfied | 1 |
|  |  | Satisfied | 2 |
|  |  | Unsatisfied | 3 |
|  |  | Very unsatisfied | 4 |
|  | Thinking about your relationship over the last three months, can you please say whether you personally agree or disagree with the following statements: | |  |
| Q76.5 | My partner gives me emotional support | I strongly agree | 1 |
|  |  | I agree | 2 |
|  |  | I disagree | 3 |
|  |  | I strongly disagree | 4 |
| Q76.6 | I give emotional support to my partner | I strongly agree | 1 |
|  |  | I agree | 2 |
|  |  | I disagree | 3 |
|  |  | I strongly disagree | 4 |
| Q76.7 | My partner usually asks my advice when they have a problem | I strongly agree | 1 |
|  |  | I agree | 2 |
|  |  | I disagree | 3 |
|  |  | I strongly disagree | 4 |
| Q76.8 | I usually ask my partner for advice when I have a problem | I strongly agree | 1 |
|  |  | I agree | 2 |
|  |  | I disagree | 3 |
|  |  | I strongly disagree | 4 |
| Q76.9 | I am fearful of my partner when he/she becomes angry | I strongly agree | 1 |
|  |  | I agree | 2 |
|  |  | I disagree | 3 |
|  |  | I strongly disagree | 4 |
| **Trust** | | | |
|  | Thinking about your relationship over the last 3 months, can you please say whether you personally agree or disagree with the following statements: | |  |
| Q77.1 | I feel my partner tries to be honest with me | I strongly agree | 1 |
|  |  | I agree | 2 |
|  |  | I disagree | 3 |
|  |  | I strongly disagree | 4 |
| Q77.2 | I try to be honest with my partner | I strongly agree | 1 |
|  |  | I agree | 2 |
|  |  | I disagree | 3 |
|  |  | I strongly disagree | 4 |
| Q77.5 | My partner gets angry if I speak to someone of the opposite sex | I strongly agree | 1 |
|  |  | I agree | 2 |
|  |  | I disagree | 3 |
|  |  | I strongly disagree | 4 |
| **Communication** | | | |
|  | Thinking about your relationship over the last 3 months, can you please say whether you personally agree or disagree with the following statements: | |  |
| Q78.1 | I can talk to my partner about my interests and opinions | I strongly agree | 1 |
|  |  | I agree | 2 |
|  |  | I disagree | 3 |
|  |  | I strongly disagree | 4 |
| Q78.2 | My partner can talk to me about their interests and opinions | I strongly agree | 1 |
|  |  | I agree | 2 |
|  |  | I disagree | 3 |
|  |  | I strongly disagree | 4 |
| Q78.3 | I am comfortable expressing my feelings and desires to my partner | I strongly agree | 1 |
|  |  | I agree | 2 |
|  |  | I disagree | 3 |
|  |  | I strongly disagree | 4 |
| Q78.4 | My partner is comfortable expressing their feelings and desires to me | I strongly agree | 1 |
|  |  | I agree | 2 |
|  |  | I disagree | 3 |
|  |  | I strongly disagree | 4 |
| Q78.5 | I make time when my partner wants to talk | I strongly agree | 1 |
|  |  | I agree | 2 |
|  |  | I disagree | 3 |
|  |  | I strongly disagree | 4 |
| Q78.6 | My partner makes time when I want to talk | I strongly agree | 1 |
|  |  | I agree | 2 |
|  |  | I disagree | 3 |
|  |  | I strongly disagree | 4 |
| Q78.9 | When we disagree, I try to see my partner’s point of view | I strongly agree | 1 |
|  |  | I agree | 2 |
|  |  | I disagree | 3 |
|  |  | I strongly disagree | 4 |
| Q78.10 | When we disagree, my partner tries to see my point of view | I strongly agree | 1 |
|  |  | I agree | 2 |
|  |  | I disagree | 3 |
|  |  | I strongly disagree | 4 |
| Q78.11 | I feel comfortable talking to my partner about sex | I strongly agree | 1 |
|  |  | I agree | 2 |
|  |  | I disagree | 3 |
|  |  | I strongly disagree | 4 |
| Q78.12 | My partner feels comfortable talking to me about sex | I strongly agree | 1 |
|  |  | I agree | 2 |
|  |  | I disagree | 3 |
|  |  | I strongly disagree | 4 |
| **Decision-making** | | | |
|  | Thinking about your relationship over the last 3 months, can you please say what you think is most likely to happen in the following situations: | |  |
| Q81.1 | When we disagree… | My partner usually gets his/her way | 1 |
|  |  | I usually get my way | 2 |
|  |  | We usually compromise | 3 |
|  |  | We each get our way about half of the time | 4 |
| Q81.2 | *If in a relationship and answer 1 or more to ‘How many children up to the age of 18 are you responsible for?’:*  When decisions have to be made about our children’s schooling (e.g. whether/where to send to school, purchasing equipment/uniform)… | I always/usually make the decision by myself | 1 |
|  |  | We usually make the decisions together/share decision making | 2 |
|  |  | My partner always/usually makes the decision by himself/herself | 3 |
|  |  | N/A | 96 |
| Q81.3 | *If in a relationship and answer 1 or more to ‘How many children up to the age of 18 are you responsible for?’:*  When decisions have to be made about our children’s healthcare (e.g. taking child for a vaccination, to a clinic, to get medicine)… | I always/usually make the decision by myself | 1 |
|  |  | We usually make the decisions together/share decision making | 2 |
|  |  | My partner always/usually makes the decision by himself/herself | 3 |
|  |  | N/A | 96 |
| Q81.4 | When decisions have to be made about making large household purchases such as furniture, land or animals… | I always/usually make the decision by myself | 1 |
|  |  | We usually make the decisions together/share decision making | 2 |
|  |  | My partner always/usually makes the decision by himself/herself | 3 |
|  |  | N/A | 96 |
| Q81.6 | Do you feel that you can refuse to have sex with your partner if you do not feel like it? | Yes | 1 |
|  |  | No | 0 |
|  |  | Do not know | 99 |
| **Responding to IPV and/or taking part in activism to prevent IPV**  *For all respondents:*  Different communities deal with violence in different ways. I would like to understand how your community deals with violence. Many times in our neighbourhoods we know about or hear when there is fighting between partners. It is sometimes difficult to know what to do, if anything, in response. We’d like to know how you have responded in the last 3 months, and remember there are no right or wrong answers. | | | |
|  | In the last 3 months: | |  |
|  | Q82. Have you seen or heard a man using physical violence against his wife/partner in your community? | Yes | 1 |
|  |  | No | 0 |
|  | *Q83. If yes:*  Did you do any of the following to help?  [PLEASE SELECT ALL THAT APPLY] | I did not do anything *[can’t be selected in combination with other options]* | Tick boxes |
|  |  | Intervened to stop the violence |  |
|  |  | Gathered other people in the community to help |  |
|  |  | Informed a relative, friend, neighbour |  |
|  |  | Informed a SASA! Together community activist |  |
|  |  | Informed a ssenga or elder |  |
|  |  | Informed a LC or police, health care provider or any other authority |  |
|  |  | Talked to the woman afterwards |  |
|  |  | Talked to the man afterwards |  |
|  |  | Spoke out against violence in the community |  |
|  |  | Other (specify) |  |
|  |  | Prefer not to say |  |
|  | *If other:* please specify | [x] | Free text |
|  | I will now ask you to imagine certain situations that people in this community sometimes find themselves in. I will ask you to imagine what you think might happen in these situations. Remember there are no right or wrong answers and you may choose to skip any question. | |  |
|  | Q84. Imagine that Mary, a woman in your community, was being hit by her husband. Do you think she should tell anyone about it? | Yes, definitely | 1 |
|  |  | Probably yes, but unsure | 2 |
|  |  | Probably no, but unsure | 3 |
|  |  | Definitely not | 4 |
|  | Q85. Now imagine that Mary’s neighbours overheard the situation. Do you think they *should* take any actions to help Mary? | Yes, definitely | 1 |
|  |  | Probably yes, but unsure | 2 |
|  |  | Probably no, but unsure | 3 |
|  |  | Definitely not | 4 |
|  | Q86. Thinking about the community you live in, how likely do you think it is that Mary’s neighbours *would* try to help her? | Very likely | 1 |
|  |  | Quite likely | 2 |
|  |  | Not very likely | 3 |
|  |  | Very unlikely | 4 |
|  | Q87. Now imagine that you were Mary’s neighbor, and you overheard the situation. Which of the following actions (if any) do you think you would take?  [READ OUT OPTIONS AND SELECT ALL THAT APPLY] | I would not do anything *[can’t be selected in combination with other options]* | Tick boxes  ‘Other, specify’ is free text |
|  |  | I would intervene myself |  |
|  |  | I would gather other people in the community to help |  |
|  |  | I would inform a relative, friend, neighbour |  |
|  |  | I would inform a SASA! Together community activist |  |
|  |  | I would inform a ssenga or elder |  |
|  |  | I would inform a LC, police, health care provider or any other authority |  |
|  |  | I would talk to the woman after the violence |  |
|  |  | I would talk to the man after the violence |  |
|  |  | I would speak out against violence in the community/become a SASA! Together activist |  |
|  |  | Other |  |
|  |  | Do not know |  |
|  | *If other:* please specify | [x] |  |
| **Activism:**  Thank you for taking the time to answer so many questions about your experiences. Before we finish, I just have a few more questions about other ways you may have participated in your community in the past 3 months. Remember there are no right or wrong answers. | | | |
|  | Q88. In the last 3 months, have you: | |  |
|  | Mobilized for a SASA! Together activity | Yes | 1 |
|  |  | No | 0 |
|  | Talked with others in the community about violence against women | Yes | 1 |
|  |  | No | 0 |
|  | Talked with others in the community about healthy relationships | Yes | 1 |
|  |  | No | 0 |
|  | Spoken out about violence in the community | Yes | 1 |
|  |  | No | 0 |
|  | Talked to a couple about healthy relationships | Yes | 1 |
|  |  | No | 0 |
|  | Role modeled positive use of power | Yes | 1 |
|  |  | No | 0 |
|  | Escorted a woman experiencing violence to services | Yes | 1 |
|  |  | No | 0 |
|  | Found out who can help women experiencing violence in my community | Yes | 1 |
|  |  | No | 0 |
| We have now finished this interview. Thank you for your time and for sharing your experiences with us. Is there anything else you would like to comment on or share with us? | | | |
| *__________________________________________________________________________________*  *__________________________________________________________________________________*  *__________________________________________________________________________________* | | | |
| *If respondent is female:*  **FINISH FOR WOMEN:**  I would like to thank you very much for helping us. I appreciate the time that you have taken. I realise that some of these questions may have been difficult for you to answer, but it is only by hearing from women themselves that we can really understand relationships and families.  In case you ever hear of a woman who needs help, here is a list of organisations that provide support, legal advice and counselling services to women in Kisinga. Please do contact them if you or any of your friends or relatives need help. Their services are free, and they will keep anything that anyone says to them private.  *If respondent is male:*  **FINISH FOR MEN:**  I would like to thank you very much for helping us. I appreciate the time that you have taken. I realise that these questions may have been difficult for you to answer, but it is only by hearing from men themselves that we can really understand relationships and families.  In case you ever hear of a man who might want support with his relationship, here is a list of organisations that provide support to men in Kisinga. Please do contact them if you or any of your friends or relatives need help or want to talk over their situation with anyone. Their services are free, and they will keep anything that anyone says to them private. | | | |
