# Supplementary material for: To what extent does Together with Gloria! expand the reach of SASA! Together community programming? A mixed methods evaluation of an edutainment intervention in Kasese, Uganda
Source: BMC Public Health. 2025 Sep 29;25:3141. doi: 10.1186/s12889-025-24178-x (PMC12481820; doi:10.1186/s12889-025-24178-x)
Supplement: Supplementary file 2 — Additional file 2. Timing and number of In-Depth Interviews (IDI) and Focus Group Discussions (FGD) conducted. Table showing the number of IDIs and FGDs conducted at Baseline, Midline and Endline [file 12889_2025_24178_MOESM2_ESM.docx]

Additional File 2: Timing and number of In-Depth Interviews (IDI) and Focus Group Discussions (FGD) conducted

|  | Baseline (6 weeks) | Midline  (19 weeks) | Endline (39 weeks) | **Total number of IDI/FGD conducted** |
| --- | --- | --- | --- | --- |
|  |  |  |  |  |
| IDI – Listeners | - | 24 | 20  (18 ‘longitudinal’ -also interviewed at Midline) | **44** |
| IDI – Community Activists | - | 15 | 12  (all 12 ‘longitudinal’ - also interviewed at Midline) | **27** |
| **IDIs - Total** | **-** | **39** | **38**  **(30 ‘longitudinal’)** | **77** |
| FGD – Listeners | 2 | - | 2 | **4** |
| FGD – Community Activists | 2 | - | 2 | **4** |
| FGD – Community Leaders | - | - | 1 | **1** |
| **FGDs** | **4** | **-** | **5** | **9** |
