# Supplementary material for: To what extent does Together with Gloria! expand the reach of SASA! Together community programming? A mixed methods evaluation of an edutainment intervention in Kasese, Uganda
Source: BMC Public Health. 2025 Sep 29;25:3141. doi: 10.1186/s12889-025-24178-x (PMC12481820; doi:10.1186/s12889-025-24178-x)
Supplement: Supplementary file 3 — Additional file 3. Characteristics associated with exposure to in-person SASA! Together programming. Table of associations between village-level and socio-demographic characteristics and exposure to in-person SASA! Together programming. [file 12889_2025_24178_MOESM3_ESM.docx]

Additional File 3: Characteristics associated with exposure to in-person *SASA! Together* programming

|  | Men  (N=512) |  |  | Women  (N=506) |  |  |
| --- | --- | --- | --- | --- | --- | --- |
|  | N | Exposed to in-person SASA! Together programming | Crude OR (95%CI) of exposure | N | Exposed to in-person SASA! Together programming | Crude OR (95%CI) of exposure |
| **Village-level characteristics** |  |  |  |  |  |  |
| Rural/Urban |  |  |  |  |  |  |
| *Rural* | 354 | 54 (15%) | - | 334 | 67 (20%) | - |
| *Urban* | 158 | 23 (15%) | 0.95 (0.48 – 1.88) | 172 | 14 (8%) | 0.35 (0.18 – 0.70) |
|  |  |  |  |  |  |  |
| Village has a SASA! Together CA? |  |  |  |  |  |  |
| *No CA/inactive* | 141 | 15 (11%) | - | 169 | 36 (21%) | - |
| *Active CA* | 371 | 62 (17%) | 1.69 (0.77 - 3.69) | 337 | 45 (13%) | 0.57 (0.20 – 1.60) |
|  |  |  |  |  |  |  |
| Distance from other villages |  |  |  |  |  |  |
| *<3 hour walk from other villages* | 461 | 74 (16%) | - | 397 | 60 (15%) | - |
| *3-4 hour walk from other villages* | 51 | 3 (6%) | 0.33 (0.22 – 0.48) | 109 | 21 (19%) | 1.34 (0.48 – 3.77) |
|  |  |  |  |  |  |  |
| **Individual-level characteristics** |  |  |  |  |  |  |
| Age-group |  |  |  |  |  |  |
| *18-25 years* | 176 | 20 (11%) | - | 128 | 27 (21%) | - |
| *26-35 years* | 144 | 18 (13%) | 1.11 (0.54 – 2.28) | 160 | 19 (12%) | 0.50 (0.18 – 1.44) |
| *36-50 years* | 132 | 33 (25%) | 2.60 (1.85 – 3.65) | 160 | 27 (17%) | 0.76 (0.33 – 1.72) |
| *51-90 years* | 60 | 6 (10%) | 0.87 (0.36 – 2.09) | 58 | 8 (14%) | 0.60 (0.28 – 1.26) |
|  |  |  | *1.21 (1.08 – 1.37)* |  |  | *0.89 (0.66 – 1.19)* |
| Education |  |  |  |  |  |  |
| *Primary or none* | 289 | 36 (12%) | - | 391 | 55 (14%) | - |
| *Secondary or higher* | 222 | 41 (18%) | 1.59 (0.97 – 2.61) | 115 | 26 (23%) | 1.78 (1.13 – 2.82) |
|  |  |  |  |  |  |  |
| Current relationship status |  |  |  |  |  |  |
| *Regular partner* | 342 | 53 (15%) | - | 385 | 68 (18%) | - |
| *No regular partner* | 170 | 24 (14%) | 0.90 (0.56 – 1.43) | 121 | 13 (11%) | 0.56 (0.36 – 0.88) |
|  |  |  |  |  |  |  |
| Number of children <18 years that respondent is responsible for |  |  |  |  |  |  |
| *0* | 193 | 21 (11%) | - | 204 | 51 (25%) | - |
| *1-3* | 209 | 40 (19%) | 1.94 (1.19 – 3.16) | 218 | 24 (11%) | 0.37 (0.21 – 0.65) |
| *4+* | 110 | 16 (15%) | 1.39 (0.71 – 2.73) | 84 | 6 (7%) | 0.23 (0.16 – 0.33) |
|  |  |  | *1.22 (0.91 – 1.64)* |  |  | 0.43 (0.32 – 0.58) |
| Have a physical disability (excluding hearing loss) |  |  |  |  |  |  |
| *No* | 473 | 72 (15%) | - | 478 | 75 (16%) | - |
| *Yes* | 39 | 5 (13%) | 0.82 (0.36 – 1.87) | 28 | 6 (21%) | 1.47 (0.49 – 4.40) |
|  |  |  |  |  |  |  |
| Have some difficulty hearing |  |  |  |  |  |  |
| *No* | 487 | 74 (15%) | - | 487 | 77 (16%) | - |
| *Yes* | 25 | 3 (12%) | 0.76 (0.27 – 2.16) | 19 | 4 (21%) | 1.42 (0.66 – 3.05) |
|  |  |  |  |  |  |  |
| Religion |  |  |  |  |  |  |
| *Catholic* | 196 | 31 (16%) | - | 242 | 29 (12%) | - |
| *Anglican* | 245 | 32 (13%) | 0.80 (0.49 – 1.31) | 210 | 47 (22%) | 2.12 (1.20 – 3.73) |
| *Other* | 71 | 14 (20%) | 1.31 (0.54 – 3.14) | 54 | 5 (9%) | 0.75 (0.27 – 1.05) |
|  |  |  |  |  |  |  |
| Own a radio |  |  |  |  |  |  |
| *No* | 218 | 28 (13%) | - | 271 | 33 (12%) | - |
| *Yes* | 294 | 49 (17%) | 1.36 (0.82 – 2.25) | 234 | 47 (20%) | 1.81 (0.98 – 3.35) |
|  |  |  |  |  |  |  |
| Radio listening frequency |  |  |  |  |  |  |
| *Less often than most days* | 280 | 31 (11%) | - | 382 | 66 (17%) | - |
| *Most days* | 227 | 43 (19%) | 1.88 (0.93 – 3.81) | 115 | 15 (13%) | 0.72 (0.31 – 1.69) |
|  |  |  |  |  |  |  |
